# A retrospective study of eating and psychosocial problems in patients with hepatic glycogen storage diseases and idiopathic ketotic hypoglycemia: Towards a standard set of patient‐reported outcome measures

**DOI:** 10.1002/jmd2.12253

**Published:** 2021-10-10

**Authors:** Annieke Venema, Fabian Peeks, Marlies de Bruijn‐van der Veen, Foekje de Boer, Marieke J. Fokkert‐Wilts, Charlotte M. A. Lubout, Bibi Huskens, Eric Dumont, Sandra Mulkens, Terry G. J. Derks

**Affiliations:** ^1^ Department of Metabolic Diseases, Beatrix Children's Hospital, University Medical Centre Groningen University of Groningen Groningen The Netherlands; ^2^ SeysCentra, Center for Paediatric Eating Problems and Incontinence Malden The Netherlands; ^3^ Department of Psychiatry and Neuropsychology, Faculty of Health, Medicine, and Life Sciences Maastricht University Maastricht The Netherlands; ^4^ Department of Clinical Psychological Science, Faculty of Psychology and Neuroscience Maastricht University Maastricht The Netherlands

**Keywords:** Avoidant/Restrictive Food Intake Disorder, dietary treatment, eating problems, glycogen storage disease, patient‐reported outcome measures, quality of life

## Abstract

There is a paucity in literature on eating and psychosocial problems in patients with hepatic glycogen storage disease (GSD) and idiopathic ketotic hypoglycemia (IKH), problems that can greatly affect quality of life. This is a monocentre, retrospective, observational mixed method study of patients with hepatic GSD or IKH treated at the Beatrix Children's Hospital Groningen, who had been referred to SeysCentra, a specialist centre for the treatment of eating problems. Additionally, a systematic literature review has been performed to identify instruments to quantify patient‐reported outcome measures of psychosocial problems in hepatic GSD patients. Sixteen patients from 12 families were included with ages ranging between 3 and 24 years. Five out of sixteen patients were diagnosed with Avoidant/Restrictive Food Intake Disorder and six patients showed characteristics of this disorder. Fourteen patients experienced sleeping problems, and 11 out of 12 parent couples experienced stress about the illness of their child. We subsequently identified 26 instruments to quantify patient‐reported outcome measures for GSD patients. This study demonstrates that GSD patients can develop Avoidant/Restrictive Food Intake Disorder influencing quality of life at multiple domains. The identification of instruments to assess psychosocial wellbeing is an important step towards a standard set of patient‐reported outcome measures.

**Abbreviations**
ARFID: Avoidant/Restrictive Food Intake Disorder
DSM‐5: Diagnostic and Statistical Manual of Mental Disorders, Fifth Edition
GSD: glycogen storage disease
IEMs: inborn errors of metabolism
IKH: idiopathic ketotic hypoglycemia
INA: information not available
n/a: not applicable
NG: nasogastric
PEG: percutaneous endoscopic gastrostomy
PROMs: patient‐reported outcome measures
QoL: quality of life
UCCS: uncooked cornstarch
UMCG: University Medical Center Groningen

**SYNOPSIS**

This study demonstrates that hepatic glycogen storage disease patients can develop Avoidant/Restrictive Food Intake Disorder and that their quality of life can be influenced by multiple factors.

## 1 INTRODUCTION

Hepatic glycogen storage disease (GSD) is a group of rare inborn errors of metabolism (IEMs) characterised by an abnormal synthesis or degradation of glycogen, because of a missing enzyme or transporter. Clinically, patients are characterised by reduced fasting tolerance, failure to thrive and hepatomegaly. Biochemical hallmarks of the diseases are GSD subtype specific and include ketotic and hypoketotic hypoglycaemia.^1^, ^2^, ^3^, ^4^, ^5^ The most frequent cause of hypoglycaemia in childhood is idiopathic ketotic hypoglycemia (IKH), which is a diagnosis of exclusion.^6^ The primary aim of dietary treatment in both GSD patients and IKH patients is to maintain euglycemia. In GSD, equally important and related to glycaemic control is to prevent secondary metabolic derangements and long‐term complications. Treatment modalities may include frequent feeds, daytime complex carbohydrates, limitation of mono‐ and disaccharides and a nocturnal continuous supply of carbohydrates in the form of either a late‐evening meal (ie, uncooked cornstarch (UCCS) or modified cornstarch (Glycosade®) or gastric drip feeding administered via nasogastric (NG) tube or percutaneous endoscopic gastrostomy (PEG). Additionally, a high protein diet is beneficial for patients with ketotic GSD subtypes.^1^, ^2^, ^3^, ^4^, ^5^, ^7^

Avoidant/Restrictive Food Intake Disorder (ARFID) is a recently acknowledged feeding or eating disorder, included in the Diagnostic and Statistical Manual of Mental Disorders, fifth edition (DSM‐5)^8^ and was previously referred to as Feeding Disorder of Infancy or Early Childhood (DSM‐IV).^9^ This disorder is characterised by eating too little and/or too selectively, associated with weight loss, poor growth, nutritional deficiencies, the need for supplementary enteral feeding or oral nutritional supplements and/or impaired psychosocial functioning. The DSM‐5 provides three representations of ARFID, namely (1) a lack of interest in food or eating, (2) an avoidance of food due to its sensory characteristics, and (3) a concern about the aversive consequences of eating the food.^10^, ^11^ It has been found that the health‐related quality of life (QoL) is decreased in patients with ARFID,^12^ which emphasises the importance of timely diagnosis and treatment. A retrospective case‐control study of 712 children demonstrated, that ARFID patients are more likely to have an underlying medical disorder.^13^

Psychosocial problems have been reported in patients with hepatic GSD. A prospective study of 31 youth with GSD I reported reduced QoL and less independently functioning. Moreover, it was recognised that their parents experienced more stress compared to parents of healthy control children.^14^ In a study that interrogated 64 individuals with GSD with questionnaires and interviews, it was observed that GSD patients demonstrated more dysfunctional attitudes towards food, with a lower body esteem.^15^ Furthermore, GSD patients more often displayed a selective food intake and increased fear of feeding. It seems evident that, especially GSD I patients may be at a higher risk of developing feeding disorders, as some patients even demonstrate complete food refusal.^16^

Recently, we have shown that young GSD patients and their parents experience many safety issues and acute complications accompanying their disease and dietary treatment.^17^ However, there is paucity in literature on feeding/eating disorders and psychosocial problems in patients with hepatic GSD. Therefore, we performed two studies: first, we carried out an observational, retrospective systematic analysis of clinical characteristics, eating problems and psychosocial problems in a subset of GSD patients, who were referred to a specialist centre because of the severity of their eating problems. Subsequently, we performed a systematic literature review to identify instruments to quantify patient‐reported outcome measures (PROMs) in GSD patients.

## 2 METHODS

### 2.1 Ethical approval

The Medical Ethical Committee of the University Medical Center Groningen (UMCG) stated that the Medical Research Involving Human Subjects Act was not applicable and that official study approval by the Medical Ethical Committee was not required (METc 2019/119). Patients with GSD or IKH were followed at the Section of Metabolic Diseases, Beatrix Children's Hospital, UMCG, an endorsed centre of expertise for GSD patients. For the retrospective part of this study, additional written informed consent to study the patient file was obtained from the patients who had been treated at SeysCentra, a specialised centre for the multidisciplinary treatment of severe eating problems.

### 2.2 Study design

First, we performed a monocentre retrospective, observational mixed method study of medical files of hepatic GSD patients before October 1, 2020. Longitudinal data were retrieved from both the UMCG paper and electronic medical files, and from the files at SeysCentra, a specialised centre in paediatric feeding and eating issues. We subsequently performed a systematic review of literature to list the instruments to quantify PROMs in patients with hepatic GSD.

### 2.3 Study of medical files

#### 2.3.1 Participants

Data were studied from all patients followed at the UMCG in the Netherlands, who had been referred to SeysCentra, and who either had a genetically or enzymatically confirmed diagnosis of hepatic GSD, or fulfilled the criteria of the diagnosis IKH.

#### 2.3.2 Data

From the paper and electronic files from all included patients at UMCG and from all patients treated at SeysCentra, retrospective data were studied by one researcher. With the help of the co‐authoring panel of experts in hepatic GSD and eating disorders, a checklist was made including following themes: demographic information, medical history and complications, social (family, school) items, eating problems, psychosocial and school problems. Data of meals per day, dietary restrictions, supplements and late evening meal of dietary management was noted as the advised dietary treatment at time of the research. Regarding eating problems, data collection focused on qualitative descriptions of problems with certain meals or products, problems with eating transitions, problems with the prescribed GSD diet, aversive experiences related to eating, behavioural observations of food refusal, lack of appetite or interest in food, sensory aversion to food, tension around meal times, delaying eating, the start of the eating problems and eating disorders (including ARFID) according to DSM‐5 criteria^18^, ^19^ as mentioned in the files. Considering psychosocial problems, emphasis was on self‐image, being bullied, problems in social life, attachment problems, social consequences of the GSD diet, dependence of parents, problems at school, parent/family situation, parental stress and the treatment at SeysCentra. It was investigated whether any of the above‐mentioned parameters appeared in a patient's file from the UMCG or SeysCentra. To this end, all notes, letters and documents from doctors and paramedics (dieticians, psychologists, nurses, remedial educationalists and social workers) were systematically searched.

#### 2.3.3 Statistics

The data were analysed in both a quantitative and qualitative manner. Data from the group of patients were mostly analysed quantitatively, and individual patient data were analysed qualitatively. Descriptive statistics were used, as numbers and percentages.

#### 2.3.4 Systematic review

A systematic review of literature was performed by one researcher about psychosocial problems in patients with hepatic GSD, to investigate the used instruments for measuring psychosocial outcomes. The research question was: ‘Which instruments are used to measure psychosocial outcomes in patients with hepatic glycogen storage disease?’ A detailed presentation of the search strategy including the Preferred Reporting Items for Systematic Review and Meta‐Analysis Protocols (PRISMA‐P) 2015 checklist, is presented as a supplementary file. In brief, the inclusion criteria were: GSD 0, Ia, Ib, III, IV, VI and IX, all ages, sex, race, ethnicity, the use of questionnaires, inventories or assessments and QoL or psychosocial outcomes. The exclusion criteria were: no English article available, other types of GSD.

## 3 RESULTS

Between June 2012 and December 12, 2019 out of 201 GSD patients and 4 IKH patients (nine males, seven females) from 12 families followed at UMCG were referred to SeysCentra. The GSD patients were diagnosed with GSD Ia (*n* = 2), Ib (*n* = 2), IIIa (*n* = 4), and IX (*n* = 4). Median age at referral to SeysCentra was 6.5 years (range: 2.2‐23.2 years). Table 1 summarises the general patient characteristics, diets, symptoms and complications of the patient cohort. Thirteen patients used UCCS or Glycosade® and 14 of the 16 patients had an NG tube of a PEG tube. Seven patients had experienced several chronic complications of their diseases, such as growth retardation, hypertriglyceridaemia or muscle weakness.

**TABLE 1:** General patient characteristics

							At time of research									
Patient number	Gender	Age at time of research (years)	GSD type	Age at diagnosis GSD or IKH (years)	Treated at SeysC	Age at referral SeysC (years)	Meals per day	Restrictions	Supplements	Late evening meal	Night feeding	Glycosade® or UCCS oral	Tube‐feeding	PEG/NGT longitudinal	Long term complications	Genotype
1	M	7	IIIa	1	No	6	6	Sucr, fruct, lac	Protein, phlexyvits	Yes	No	No	Dependent	Both	Ventricular hypertrophy	Homozygote c.1283+1G>A AGL gene
2^a^	M	4	IX	1	Yes	3	10	Sucr	Protein, UCCS	Yes	No	Yes	Yes, for eating problem	Both	No	Hemizygote c.3614C>T PHKA2 gene
3	M	8	Ib	0	No	7	8	Sucr, fruct, lac	Protein	Yes	Yes	Yes	Period of night feeding	NGT	Growth retardation, hyperuricemia, pancreatitis, neutropenia, IBD	Homozygote C1042_1043delCT
4^a^	F	5	IKH	2	Yes	4	6	Mono, disach	UCCS, protein	Yes	No	Yes	Yes, for eating problem	Both	No	N/A
5^a^	M	3	IKH	0	Yes	2	6	No	UCCS, protein	Yes	No	Yes	Yes, for eating problem	NGT	Growth retardation, hypertriglyceridaemia	N/A
6	M	9	IKH	5	No	7	6	No	UCCS, protein	Yes	No	Yes	No	No	No	N/A
7	F	7	IKH	4	No	5	6	No	UCCS, protein	Yes	No	Yes	Temporary, for eating problem	NGT	No	N/A
8	M	9	IX	1	Yes	4	6	Sucr	Glycosade®, protein	Yes	No	Yes	Yes	NGT	Growth retardation, hypertriglyceridaemia	Enzymatically confirmed
9	M	4	IX	0	No	4	7	Sucr	Glycosade®, protein	Yes	No	Yes	Yes	NGT	No	Enzymatically confirmed
10	F	24	IIIa	0	No	23	7	Sucr	Glycosade, protein	Yes	No	Yes	Yes, temporary at night	Both	Growth retardation, cardiomyopathy, hyperuricemia, hyperlipidemia	Homozygote c.643G>A AGL gene
11	F	17	Ia	1	No	13	2 h	Sucr, fruc, lac	UCCS, Glycosade®	Yes	No	Yes	Yes, period dependent	Both	No	Homozygote c.1118C.T G6PC gene
12	M	14	IX	3	Yes	6	INA	Sucr	Glycosade®	Yes	No	Yes	No	No	Growth retardation	Hemizygote c.3614C>T PHKA2 gene
13^a^	F	8	IIIa	1	Yes	7	8	Fruct, lact	Glycosade®, protein	Yes	Yes	Yes	Yes, night feeding	NGT	Ventricular hypertrophy, distal muscle weakness	Heterozygote c.847‐2A>T and c.721T>C AGL gene
14	M	9	Ia	0	No	9	11	Sucr, fruct, lac	Glycosade®, protein	Yes	Yes	Yes	Yes	NGT	Growth retardation, osteopenia, hypertriglyceridaemia	Homozygote c.888G>T G6PC gene
15^a^	F	14	IIIa	12	Yes	13	7	Sucr	UCCS, protein	Yes	Yes	No	Yes	PEG	Growth retardation, distal muscle weakness	Homozygote c.1020del AGL gene
16	F	14	Ib	0	No	13	2 h	Sucr, fruct, lac	Glycosade®, protein	No	Yes	No	Yes	NGT	Growth retardation, hypertriglyceridaemia, neutropenia	Homozygote c.1211‐1212delCT

*Note*: No distinction was made in protein supplements. 2 h = every 2 hours, disach = disaccharides, F = female, fruct = fructose, hypo = hypoglycaemia, IBD = inflammatory bowel disease, INA = information not available, lac = lactose, M = male, N/A not applicable, =NGT = nasogastric tube feeding, PEG = percutaneous endoscopic gastrostomy, SeysC = SeysCentra, sucr = sucrose, UCCS = uncooked cornstarch.

^a^ Diagnosed with ARFID.

### 3.1 Problems with the GSD diet

Twelve out of the 14 patients who used UCCS experienced problems with it. Four patients had trouble with the introduction, four patients experienced diarrhoea and four patients complained about the bad taste. Other problems were intolerance (*n* = 2), vomiting (*n* = 2), abdominal pain (*n* = 2) and increased defecation frequency (*n* = 1). Seven out of the 12 patients who had used Glycosade® reported the following problems: diarrhoea (*n* = 3), vomiting (*n* = 3), trouble with introduction (*n* = 2), intolerance (*n* = 2) and bad taste (*n* = 2). Fourteen patients used protein supplementation of whom five patients reported the following problems: bad taste (*n* = 3), trouble with the introduction (*n* = 1), intolerance (*n* = 1) and changed defecation (*n* = 1).

### 3.2 Eating problems

Table 2 summarises the eating problems identified in this cohort. All referred patients showed a form of selective eating and food refusal behaviour. In the categories ‘selective eating’ and ‘tension around meal times’ two patients (Patients 1 and 11, see Table 1) were not taken into account because they were fully dependent on tube feeding, of whom Patient 1 (see Table 1) had never tried to eat food. Seven patients experienced a period of complete food refusal, and seven patients did not eat age appropriately. In four patients it was mentioned that there was trouble with introducing new food, three patients had trouble with discontinuing tube feeding and five patients did not eat independently.

**TABLE 2:** Eating problems in the GSD and IKH patients

Eating problem	Number of patients	%	Expressed in problems with	Number of patients	%
Selective eating	14/14^a^	100	Breakfast	9/14	64
			Lunch	3/14	21
			Snacks	3/14	21
			Warm food	8/14	57
			Late evening meal	8/14	57
			Breast feeding	1/14	7
			Follow‐on milk	1/14	7
			Dairy products	5/14	36
			Cereal products	8/14	57
			Potatoes	2/14	14
			Vegetables	7/14	50
			Fruit	4/14	29
			Meat/fish	5/14	36
			Eggs	2/14	14
Aversive symptoms during eating	13/16	81	Hypersensitivity of mouth/negative experiences in mouth	8/13	62
			Fear of symptoms	3/13	23
			Vomiting	6/13	46
			Gag reflex	6/13	46
			Choking	1/13	8
			Stomach ache	5/13	38
			Nausea	4/13	31
Food refusal behaviour	14/15^b^	93	Resistance while eating/conflict	13/14	93
			Indicating not to be hungry	3/14	21
			Angry during eating	5/14	36
			Turning head away/not taking a bite	4/14	29
			Pushing the spoon away	3/14	21
			Not opening mouth	1/14	7
			Asking for tube feeding	3/14	21
			Eating little food	10/14	71
			Taking small bites	2/14	14
			Working food out of mouth	5/14	36
			Crying	5/14	36
			Withdrawal behaviour	1/14	7
			Negotiating	4/14	29
			Playing with food	1/14	7
			Complete food refusal	7/14	50
Lack of appetite or interest in food	11/16	69	Eating little food	3/11	27
			Not wanting to try new food	3/11	27
			Not hungry	5/11	45
			Feeling full	6/11	55
			Eating slowly	6/11	55
			Forgetting to eat	2/11	18
			Must be urged to eat	10/11	91
Sensory aversion to food	7/15^b^	47	Taking small bites	4/7	57
			Working food out mouth	3/7	43
			Not wanting to swallow/chew	3/7	43
			Eating slowly	4/7	57
			Eating only ground food	2/7	29
Tension around meal times	9/14	64	Restlessness	3/9	33
			Arguments and stress	8/9	89
Other			Procrastination of eating	3/14	21
			Distracted while eating	6/14	43

^a^ *n* = 2 were dependent on tube feeding and were not included in this variable.

^b^ *n* = 1 was dependent on tube feeding and never tried food and was not included in these variable.

In four patients, the eating problems had started before the first year of life. Crucial moments that luxated or worsened the eating problems included: being dependent on tube feeding, traumatic experiences due to sickness or tube insertion, start of the dietary management, restriction of simple carbohydrates in diet, acute hospitalisations and intercurrent illnesses. At least in six patients it was mentioned that eating problems worsened when the patient was sick, whereas three patients experienced worsened eating problems when stressed.

### 3.3 SeysCentra and ARFID

Out of the 16 patients that were referred from UMCG to SeysCentra, only seven patients were eventually treated at SeysCentra, while one patient was treated twice. The most often mentioned reason for not starting treatment was the fact that treatment was too time intensive. The indications for treatments were selective eating (*n* = 5), more responsibility for disease or diet (*n* = 4), trouble with the GSD diet (UCCS, Glycosade®) (*n* = 3), help with quitting tube feeding (*n* = 2), aversive symptoms (*n* = 1), eating independently (*n* = 2), eating faster (*n* = 1), education around disease (*n* = 1) and food refusal (*n* = 1). Most patients had more than one reason for indications for treatments. The median duration of treatment at SeysCentra was 9 months (range 1 week ‐ 14 months). The following treatments were mentioned: day treatment, Eye Movement Desensitization and Reprocessing, ambulatory treatment, 24‐h treatment, treatment at home and part‐time day treatment. Six of the seven patients had achieved their treatment goals.

Five out of 16 patients were diagnosed with ARFID at SeysCentra. Six patients showed characteristics of ARFID, and five patients had no characteristics of ARFID based on information in the medical files. In Table 1, the patients with the diagnosis ARFID are indicated with an asterisk. Most patients with (characteristics of) ARFID met multiple subtypes,^10^, ^11^ such as (1) avoidant or restrictive food intake due to the sensory characteristics of food (*n* = 8), (2) lack of interest in food (*n* = 7), and (3) avoidance of certain foods or eating due to fear of the aversive consequences (*n* = 1). Importantly, no characteristics of other eating disorders were found.

### 3.4 Psychosocial problems

One patient had documented problems with his/her self‐esteem and four patients compared themselves much with other people. Three patients experienced problems with their diet at school. It was found that five patients were not reaching self‐dependence in their diet or disease management as expected for age. Trouble with sleeping emerged as an important theme as well. It was found that of the 16 patients, 14 patients had problems with sleeping.

Fifteen patients went to school, one patient was too young. Eight patients who went to school experienced problems at school. Four of those had to repeat school years, three patients were absent much because of their diseases, three patients were struggling to keep up with the teaching material, three patients complained of tiredness, three patients had concentration problems. Lastly, three of the patients had trouble with their diet at school.

Parental stress was also an important theme. Eleven out of 12 parent couples experienced stress because of the condition of their child(ren) based on the medical files.

### 3.5 Systematic literature review

**FIGURE 1:**
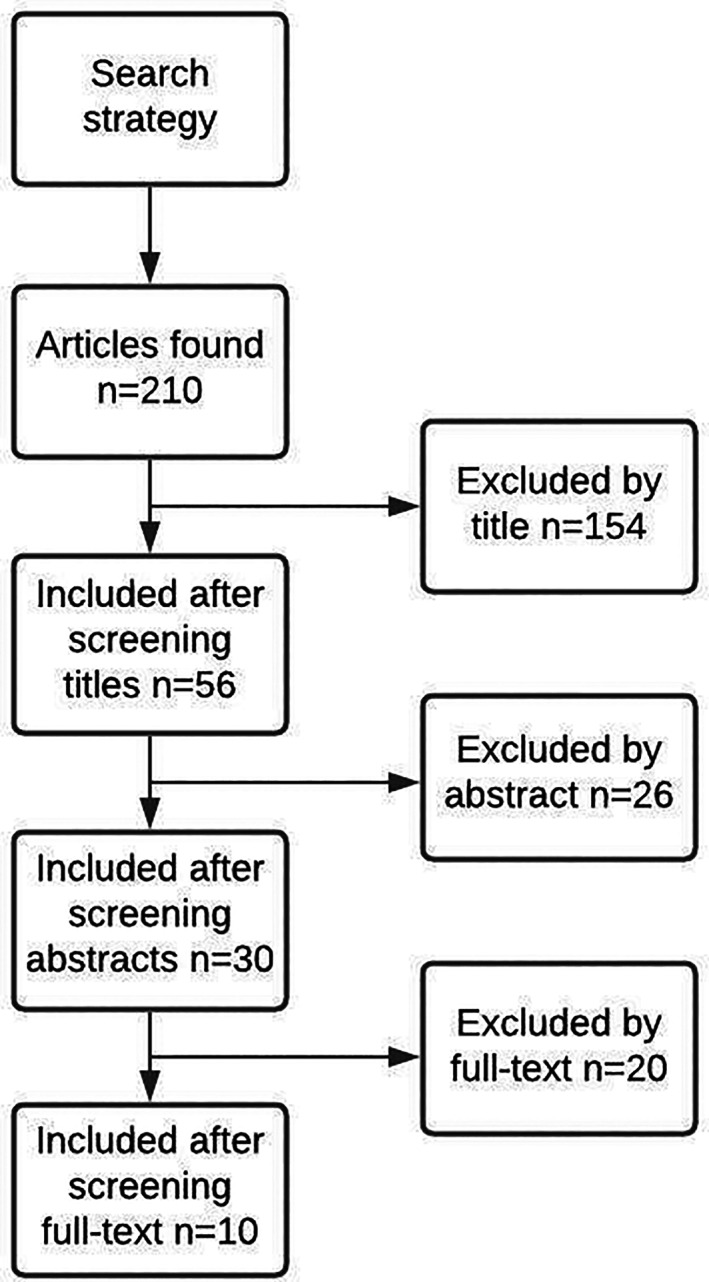
Flow chart of the search strategy

References of all included articles are presented in supplementary file. In brief, this systematic review initially included 210 references. Figure 1 presents the flowchart of the search strategy together with the steps of the systematic review. Table 3 presents the 26 instruments for PROMs applied to GSD patients reported in the literature to date

**TABLE 3:** Instruments to quantify PROMs in GSD patients

Reference	Study population	Study design	Measure instruments
Francini‐Pesenti et al., 2019^30^	34 year old patient with GSD IIIa	Case report	• Short Form Health Survey (SF‐36; Health‐related quality of life [HRQoL])
Gao et al., 2020^31^	Parents from 651 children with rare diseases, including 30 with GSD	Single centre, cross‐sectional, observational questionnaire study	• Pediatric Quality of Life Inventory™ 4.0 (PedsQL™ 4.0)
Rousseau‐Nepton et al., 2018^32^	9 adults with GSD Ia	Prospective cohort study	• Short Form Health Survey (SF‐36; Health‐related quality of life [HRQoL]) • Pittsburgh Sleep Quality Index (PSQI)
Flanagan et al., 2015^15^	64 patients > 7 years of age with GSD I, GSD IIIa, GSD VI, GSD IX, and unclassified	Single centre, cross sectional, observational questionnaire study Questionnaires on eating disorder symptoms, eating attitudes and body image	• Eating Disorders Inventory‐3 (EDI‐3) Eating Disorders Inventory‐Child (EDI‐C) • Eating Attitudes Test (EAT) • Children's Eating Attitude Test (ChEAT) • Body Esteem Scale (BES) • Body Esteem Scale for Children (BES‐C)
Steunenberg et al., 2018^17^	249 (caregivers of) patients with self‐reported hepatic GSD. Median age of 14.8 years (range: 0.5–66.1).	Questionnaire	A questionnaire was developed, with three segments: personal information, dietary management and complications
Michon et al., 2015^33^	7 GSD III patients mean age 38.7 ± 11.6 years	Pilot study Neuropsychological set of tests assessing global cognitive efficiency, executive functions, social cognition, apathy, and episodic memory.	• MINI lifetime 5.0.0: Mini International Neuropsychiatric Inventory • State and Trait Anxiety Inventory (STAI Y‐A and STAI Y‐B) • Social Adjustment Self Report (SAS‐SR)
Storch et al., 2008^14^	31 patients from 3 to 25 years old with GSD I and their parents 42 healthy individuals and their parents	Prospective. QoL ratings from the GSD types Ia and Ib sample were compared with a previously reported clinical comparison sample	• Pediatric Quality of Life Inventory (PedsQL) • Asher Loneliness Scale (ALS) • Child Behavior Checklist (CBCL) • Brief Symptom Inventory (BSI) • Pediatric Inventory for Parents (PIP) • McMaster Family Assessment Device (FAD) • AAMR Adaptive Behavior Scale‐School: Second Edition (ABS‐S2) • Sibling Relationship Questionnaire (SRQ)
Brown et al., 2015^34^	Parents of children (*n* = 22) with confirmed inherited metabolic disorder (including glycogen storage disease)	Questionnaires	• Kessler 10 Psychological Distress Scale • Parent Experience of Childhood Illness (PECI) Short Form • Family Management Measure (FaMM) • Strengths and Difficulties Questionnaire (SDQ) • Wechsler Abbreviated Scale of Intelligence (WASI) • Wechsler Preschool and Primary Scale of Intelligence Third Edition (WPPSI‐III)
Martinez et al., 2019^16^	36 patients (range, 8.0–18.7 years) with confirmed diagnoses of GSD (type Ia, Ib, III, Ixa, IXc)	Cross‐sectional, prospective study. Questionnaires, olfactory and taste performance and facial anthropometry.	• The orofacial myofunctional evaluation (AMIOFE)
Sechi et al., 2014^35^	38 patients (22 females, 16 males; 27 with GSD Ia, 11 with GSD Ib, of 16 years or older)	Assessing QoL in adult patients with GSD I.	• The Italian version of the Short Form Health Survey (SF‐36) questionnaire.

## 4 DISCUSSION

This is the first study to report the presence of (characteristics of) ARFID in a number of patients with GSD or IKH that were referred to a specialised treatment centre for paediatric eating problems (SeysCentra). In addition, multiple eating and psychosocial problems were identified in this population. These GSD and IKH patients struggled with poor appetite, selective eating, slow eating and aversive symptoms during mealtimes. We assume that the patients in our study represent a typical top of the iceberg and that eating and psychosocial problems are underexposed during regular patient care. Secondly, we have identified 26 instruments PROMs for GSD patients. The above‐mentioned problems can all have a serious effect on the QoL of these families. Therefore, awareness and multidisciplinary assessment are warranted to prevent eating and psychosocial problems in these patients.

This study builds on existing evidence of feeding difficulties in children with IEMs. Evans and co‐workers performed an observational, pilot study in 20 children with a variety of IEMs, including urea cycle defects, organic acidurias and aminoacidopathies (not phenylketonuria).^20^ Caregivers, by answering questionnaires, reported poor appetite, limited food variety and lengthy mealtimes. More recently, Bérat and co‐workers performed a retrospective descriptive study on the use of enteral tube feeding in 98 out of 190 IEMs patients, amongst whom were 23 GSD patients receiving enteral tube feeding.^21^ In this study, parents reported a decrease in oral feeding and total feeding difficulties after gastrostomy. Furthermore, parents reported an improvement in QoL after gastrostomy but eating problems and psychosocial problems were not systematically addressed. In their cross‐sectional prospective study in 36 GSD patients, Martinez and co‐workers observed eating problems in 50–72% of the patients.^16^ Previous studies have described an association between food refusal and NG tube exposure >3 months in children with various disorders,^22^ and this phenomenon was also described in a study of Dello Strologo in children with severe chronic renal failure.^23^ Hence, GSD patients can present eating disorders and psychosocial problems, especially when receiving a NG tube or gastrostomy. Food refusal can lead to life threatening situations for these patients with fasting intolerance, causing both patient and parental stress, possibly aggravating eating and psychosocial problems.

It is important to note that in the general population nearly half of children are selective eaters at some point during early childhood.^24^ Prevalence of picky eating is mostly transitory behaviour in preschool children between 0 and 4 years, corresponding with the age at which most GSD and IKH patients are usually diagnosed, which illustrates that dietary management is initiated at a vulnerable age. We cannot exclude that some eating problems can be a consequence of the dietary treatment of hepatic GSD patients. Due to frequent meals and the intake of complex carbohydrates, these patients often feel full, not hungry and lack appetite to eat. The insertions of NG tubes may have aggravated the development of eating problems.

It became apparent that 14 of the patients experienced sleeping problems. Sleeping problems are associated with poorer QoL in preschool aged children^25^ and they can be an underlying factor for mental health problems in adolescents.^26^ Sleeping problems are recognised in other chronic diseases, such as inflammatory bowel disease.^27^ In this study, sleeping problems may be a practical consequence of nocturnal dietary treatment, or due to the fear of nocturnal hypoglycaemias.

The results of the present study should be interpreted with caution, due to some important methodological limitations. Psychosocial problems have not been systematically assessed during medical follow‐up, hence assessment of the medical files may have caused certain factors being overlooked and to a certain degree of interpretation. The data in the files were originally collected for other purposes, namely the treatment and guidance of the individual patient and not for research purposes, which may have resulted in sampling bias. Related to this is the fact that part of the data were collected before the diagnosis of ARFID – with its current criteria – was included in the DSM‐5. Therefore, some of the symptoms and representations of ARFID might have been described differently in the medical files before and after this new diagnosis became customary in the field (around 2017). There was also a potential selection bias towards patients referred to a centre specialised in the treatment of eating problems. This study certainly did not cover all patients with hepatic GSD with eating and psychosocial problems and we assume that the referred patients represented the top of the iceberg, for which we obviously could not include a control group. IKH patients were also included in this cohort study. IKH is a diagnosis of exclusion with a variable presentation, but in a subset of the patients, dietary management may have similarities with care for hepatic GSD patients.^6^ Although the cohort may not be representative for the whole cohort of patients with fasting intolerance, the authors believe that important lessons can be learnt from the observations in these patients with GSD and IKH.

Dietary management is key for many IEM patients. Specifically in GSD and IKH patients, continuous nocturnal gastric drip feeding or UCCS supplementation aims to prevent hypoglycaemias and glycogenolysis‐, proteolysis‐ and lipolysis‐related metabolic decompensation. In current GSD guidelines, information and guidance is either scarce or absent regarding ARFID, other eating problems, psychosocial problems and sleeping problems.^1^, ^2^, ^3^, ^4^, ^7^ Management guidelines for GSD I patients acknowledge that biomedical targets for the management of patients should be attempted to be approached as much as closely, ‘without deterioration in QoL’.^4^ And that, ‘if psychosocial issues are apparent, the family may be referred to the clinical social worker or the child may need a full psychological evaluation’.^1^ Although outcomes for many patients with multiple IEMs have significantly improved in the last decades by dietary management and other medical‐technical interventions, it can be hypothesised that both the IEMs and the dietary management interventions are potential predisposing factors for the development of eating and psychosocial problems, and hence affecting QoL.

The recent international priority setting partnership for liver GSD acknowledged QoL as an overarching research priority.^28^ Our systematic review identified suitable instruments for quantifying PROMs, such as questionnaires to measure QoL (the Paediatric Quality of Life, suitable for patients between 2 and 18 years old and the 36‐item Short‐Form Health Survey [SF‐36] for patients older than 14 years old), or to screen for eating problems (The Eating Disorders Inventory‐3 [EDI‐3] and a special version for children; the Eating Disorders Inventory‐Child [EDI‐C]). The authors emphasise the importance of multidisciplinary assessments of these patients, however it is yet unclear what measures can be taken to prevent psychosocial problems in GSD patients.

To conclude, this study demonstrates that GSD patients can develop ARFID and that their QoL can be influenced by multiple factors. We have generated an overview of 26 instruments quantifying PROMs for GSD patients in medical literature. Next, patients and families should be involved in the selection of the outcome measures that matter the most to them, as exemplified for diabetes mellitus in collaboration with the International Consortium for Health Outcomes Measurement.^29^ Systematic, prospective evaluation can improve value and QoL for patients and families and may contribute to the active screening and prevention, diagnosis and specialised management of eating disorders and accompanying psychosocial problems.

## CONFLICT OF INTEREST

The authors declare no conflicts of interest.

## AUTHOR CONTRIBUTIONS

Terry G. J. Derks initiated this project, was involved in study design, data collection, data analysis, wrote the first and final version of the manuscript. Annieke Venema, Fabian Peeks and Sandra Mulkens were involved in study design, data collection, data analysis, and wrote the first and final manuscript. Marlies de Bruijn‐van der Veen, Bibi Huskens and Eric Dumont were involved in study design, data collection and data analysis. All other authors contributed to data collection and revised the manuscript for important intellectual content. All authors approved the final manuscript as submitted and agreed to be accountable for all aspects of the work. All authors confirm the absence of previous similar or simultaneous publications.

## PATIENT CONSENT

Written informed consent to study patient files was obtained from the patients who had been treated at SeysCentra, a specialised centre for the multidisciplinary treatment of severe eating problems.

## ETHICS APPROVAL

All procedures followed were in accordance with the ethical standards of the responsible committee on human experimentation (institutional and national) and with the Helsinki Declaration of 1975, as revised in 2000. Informed consent was obtained from all patients for being included in the study. The Medical Ethical Committee of the University Medical Centre Groningen stated that the Medical Research Involving Human Subjects Act was not applicable and that official study approval by the Medical Ethical Committee was not required (METc 2019/119). The study was approved for waived consent as it concerned retrospective, anonymous data.

## Supporting information


**Supplementary File S1** Systematic review protocol

## Data Availability

Data can be made available on a reasonable request to the authors.
