# Supplementary material for: A retrospective study of eating and psychosocial problems in patients with hepatic glycogen storage diseases and idiopathic ketotic hypoglycemia: Towards a standard set of patient‐reported outcome measures
Source: JIMD Rep. 2021 Oct 10;63(1):29–40. doi: 10.1002/jmd2.12253 (PMC8743343; doi:10.1002/jmd2.12253)
Supplement: Supplementary file 1 — Supplementary File S1 Systematic review protocol [file JMD2-63-29-s001.doc]

**Supplementary File 1: Systematic Review Protocol**

**A retrospective study of eating and psychosocial problems in patients with hepatic glycogen storage disease;**

**Towards a standard set of patient-reported outcome measures**

Annieke Venema^1^, Fabian Peeks^1^, Marlies de Bruijn-van der Veen^1^, Foekje de Boer^1^,

Marieke J.Fokkert-Wilts^1^, Charlotte M.A. Lubout^1^, Bibi Huskens^2^, Eric Dumont^2,3,4^,

*Sandra Mulkens^2,3,4^, *Terry G.J. Derks^1^

^1^ Department of Metabolic Diseases, Beatrix Children’s Hospital, University Medical Centre Groningen, University of Groningen, Groningen, The Netherlands

^2^ SeysCentra, Center for Paediatric Eating Problems and Incontinence, Malden, The Netherlands

^3^ Department of Psychiatry and Neuropsychology, Faculty of Health, Medicine, and Life Sciences, Maastricht University, Maastricht, The Netherlands

^4^ Department of Clinical Psychological Science, Faculty of Psychology and Neurosciences, Maastricht University, Maastricht, The Netherlands

* Shared last authors

**Journal:** JIMD

**Address correspondence to:**

Terry G. J. Derks, Department of Metabolic Diseases, Beatrix Children’s Hospital, University Medical Centre Groningen, University of Groningen, PO Box 30 01, 9700 RB, Groningen, The Netherlands, +31 (0)50 3611036, [t.g.j.derks@umcg.nl](mailto:t.g.j.derks@umcg.nl)

Contribution of individual authors

T.G.J.D. initiated this project, was involved in the research question, PICO and the writing the part of the systematic review.

A.V. en F.P. were involved in the research question, PICO, the execution and writing the part of the systematic review.

All other authors contributed to data collection and revised the manuscript for important intellectual content.

All authors approved the final manuscript as submitted and agreed to be accountable for all aspects of the work.

All authors confirm the absence of previous similar or simultaneous publications.

**Supplemental Data 1. Detailed presentation and assessment of the search strategy**

The PICO for this systematic review:

- Patient: patients with hepatic glycogen storage disease
- Intervention: questionnaires, inventory, assessments
- Comparison: /
- Outcome: psychosocial outcomes

The research question was: ‘Which measure instruments are used to measure psychosocial outcomes in patients with hepatic glycogen storage disease?’

Pubmed was searched using both MeSH terms and free text. The search strategy PubMed:

("Glycogen Storage Disease"[Mesh] OR glycogen storage diseas*[tiab] OR glycogenos*[tiab])

AND

("Psychosocial Functioning"[Mesh] OR "Psychological Tests"[Mesh] OR "Quality of Life"[Mesh] OR "Surveys and Questionnaires"[Mesh:NoExp] OR "Health Care Surveys"[Mesh] OR "Patient Health Questionnaire"[Mesh] OR "Self Report"[Mesh] OR "Loneliness"[Mesh] OR "Self Concept"[Mesh] OR "Adaptation, Psychological"[Mesh:NoExp] OR "psychology" [Subheading] OR esteem[tiab] OR psychosocial[tiab] OR psychological[tiab] OR (social[tiab] AND functioning[tiab]) OR (emotional[tiab] AND functioning[tiab]) OR quality-of-life[tiab] OR qol[tiab] OR hrqol[tiab] OR life-quality[tiab] OR patient-outcome*[tiab] OR patient-reported[tiab] OR self-report*[tiab] OR SF36[tiab] OR SF-36[tiab] OR short-form-36[tiab] OR questionnair*[tiab] OR mental-health[tiab] OR emotional-health[tiab])

NOT

("Animals"[Mesh] NOT "Humans"[Mesh])

The search strategy was conducted on the 12^th^ of April 2021. The inclusion criteria were:

- GSD Type 0, Ia, Ib, III, IV, VI and IX
- All ages, sex, race and ethnicity
- Articles from all years
- Use of questionnaires, inventories or assessments
- Quality of life or psychosocial outcomes
- English article available

The exclusion criteria were:

- Other types of GSD

One researcher conducted the systematic review.
Bias is not relevant, as we want to know which measuring instruments were used and not how the results were interpreted.

**PRISMA-P (Preferred Reporting Items for Systematic review and Meta-Analysis Protocols) 2015 checklist: recommended items to address in a systematic review protocol***

| Section and topic | Item No | Checklist item | Page(s) |
| --- | --- | --- | --- |
| ADMINISTRATIVE INFORMATION | | |  |
| Title: |  |  |  |
| Identification | 1a | Identify the report as a protocol of a systematic review | 5 |
| Update | 1b | If the protocol is for an update of a previous systematic review, identify as such | - |
| Registration | 2 | If registered, provide the name of the registry (such as PROSPERO) and registration number | - |
| Authors: |  |  |  |
| Contact | 3a | Provide name, institutional affiliation, e-mail address of all protocol authors; provide physical mailing address of corresponding author | 1 |
| Contributions | 3b | Describe contributions of protocol authors and identify the guarantor of the review | Supplemental data 1 |
| Amendments | 4 | If the protocol represents an amendment of a previously completed or published protocol, identify as such and list changes; otherwise, state plan for documenting important protocol amendments | - |
| Support: |  |  |  |
| Sources | 5a | Indicate sources of financial or other support for the review | - |
| Sponsor | 5b | Provide name for the review funder and/or sponsor | - |
| Role of sponsor or funder | 5c | Describe roles of funder(s), sponsor(s), and/or institution(s), if any, in developing the protocol | - |
| INTRODUCTION | | |  |
| Rationale | 6 | Describe the rationale for the review in the context of what is already known | 4, 5 |
| Objectives | 7 | Provide an explicit statement of the question(s) the review will address with reference to participants, interventions, comparators, and outcomes (PICO) | Supplemental data 1 |
| METHODS | | |  |
| Eligibility criteria | 8 | Specify the study characteristics (such as PICO, study design, setting, time frame) and report characteristics (such as years considered, language, publication status) to be used as criteria for eligibility for the review | 6, 7, supplemental data 1 |
| Information sources | 9 | Describe all intended information sources (such as electronic databases, contact with study authors, trial registers or other grey literature sources) with planned dates of coverage | Flowchart (figure 1), supplemental data |
| Search strategy | 10 | Present draft of search strategy to be used for at least one electronic database, including planned limits, such that it could be repeated | Flowchart (figure 1), supplemental data |
| Study records: |  |  |  |
| Data management | 11a | Describe the mechanism(s) that will be used to manage records and data throughout the review | - |
| Selection process | 11b | State the process that will be used for selecting studies (such as two independent reviewers) through each phase of the review (that is, screening, eligibility and inclusion in meta-analysis) | 7 |
| Data collection process | 11c | Describe planned method of extracting data from reports (such as piloting forms, done independently, in duplicate), any processes for obtaining and confirming data from investigators | 7 |
| Data items | 12 | List and define all variables for which data will be sought (such as PICO items, funding sources), any pre-planned data assumptions and simplifications | Supplemental data |
| Outcomes and prioritization | 13 | List and define all outcomes for which data will be sought, including prioritization of main and additional outcomes, with rationale | Supplemental data |
| Risk of bias in individual studies | 14 | Describe anticipated methods for assessing risk of bias of individual studies, including whether this will be done at the outcome or study level, or both; state how this information will be used in data synthesis | Supplemental data |
| Data synthesis | 15a | Describe criteria under which study data will be quantitatively synthesised | - |
|  | 15b | If data are appropriate for quantitative synthesis, describe planned summary measures, methods of handling data and methods of combining data from studies, including any planned exploration of consistency (such as I^2^, Kendall’s τ) | - |
|  | 15c | Describe any proposed additional analyses (such as sensitivity or subgroup analyses, meta-regression) | - |
|  | 15d | If quantitative synthesis is not appropriate, describe the type of summary planned | Table 3 |
| Meta-bias(es) | 16 | Specify any planned assessment of meta-bias(es) (such as publication bias across studies, selective reporting within studies) | - |
| Confidence in cumulative evidence | 17 | Describe how the strength of the body of evidence will be assessed (such as GRADE) | - |

*** It is strongly recommended that this checklist be read in conjunction with the PRISMA-P Explanation and Elaboration (cite when available) for important clarification on the items. Amendments to a review protocol should be tracked and dated. The copyright for PRISMA-P (including checklist) is held by the PRISMA-P Group and is distributed under a Creative Commons Attribution Licence 4.0.**

*From: Shamseer L, Moher D, Clarke M, Ghersi D, Liberati A, Petticrew M, Shekelle P, Stewart L, PRISMA-P Group. Preferred reporting items for systematic review and meta-analysis protocols (PRISMA-P) 2015: elaboration and explanation. BMJ. 2015 Jan 2;349(jan02 1):g7647.*

**References of all included articles**

1: McMillan MF, Kraft IA, Wheeler JI Jr. Von Gierke's disease in siblings—a psychiatric case study of three siblings and review of the literature. Psychosomatics. 1966 Sep-Oct;7(5):295-8. doi: 0.1016/S0033-3182(66)72091-X. PMID: 5222533.

2: Tanner JM, Whitehouse RH, Hughes PC, Vince FP. Effect of human growth hormone treatment for 1 to 7 years on growth of 100 children, with growth hormone deficiency, low birthweight, inherited smallness, Turner's syndrome, and other complaints. Arch Dis Child. 1971 Dec;46(250):745-82. doi: 10.1136/adc.46.250.745. PMID: 5288779; PMCID: PMC1647921.

3: Gutton P. Réflexions à propos d'observations d'enfants atteints de maladie métabolique éréditaire [Reflections apropos of observations of children with hereditary metabolic diseases]. Psychiatr Enfant. 1978;21(1):26-56. French. PMID: 280915.

4: Kotz YM, Rodionov IM, Sitnikov BF, Tkhorevsky VI, Vinogradova OL. On the mechanism of an increase of muscle performance and of vasodilation during emotional stress in man. Pflugers Arch. 1978 Mar 20;373(3):211-8. doi: 10.1007/BF00580826. PMID: 277891.

5: Kadota RP, Smithson WA. Bone marrow transplantation for diseases of childhood. Mayo Clin Proc. 1984 Mar;59(3):171-84. doi: 10.1016/s0025-6196(12)60770-8. PMID: 6369012.

6: Mahoney KG. Diagnosing and managing type I and type III glycogen storage diseases. MCN Am J Matern Child Nurs. 1984 Sep-Oct;9(5):338-42. doi: 10.1097/00005721-198409000-00008. PMID: 6433137.

7: Rattenbury JM, Worthy E, Allen JC. Screening tests for glycosaminoglycans in urine: experience from regional interlaboratory surveys. J Clin Pathol. 1988 Sep;41(9):936-9. doi: 10.1136/jcp.41.9.936. PMID: 3142935; PMCID: PMC1141647.

8: Schroten H, Wendel U, Burdach S, Roesler J, Breidenbach T, Schweitzer S, Zeidler C, Welte K. Colony-stimulating factors for neutropenia in glycogen storage disease Ib. Lancet. 1991 Mar 23;337(8743):736-7. doi: 10.1016/0140-6736(91)90324-i. PMID: 1706047.

9: Schroten H, Roesler J, Breidenbach T, Wendel U, Elsner J, Schweitzer S, Zeidler C, Burdach S, Lohmann-Matthes ML, Wahn V, et al. Granulocyte and granulocyte-macrophage colony-stimulating factors for treatment of neutropenia in glycogen storage disease type Ib. J Pediatr. 1991 Nov;119(5):748-54. doi: 10.1016/s0022-3476(05)80290-2. PMID: 1719175.

10: Ishiguro A, Nakahata T, Shimbo T, Amano Y, Yasui K, Koike K, Komiyama A. Improvement of neutropenia and neutrophil dysfunction by granulocyte colony-stimulating factor in a patient with glycogen storage disease type Ib. Eur J Pediatr. 1993 Jan;152(1):18-20. doi: 10.1007/BF02072510. PMID: 7680314.

11: Talente GM, Coleman RA, Alter C, Baker L, Brown BI, Cannon RA, Chen YT, Crigler JF Jr, Ferreira P, Haworth JC, Herman GE, Issenman RM, Keating JP, Linde R, Roe TF, Senior B, Wolfsdorf JI. Glycogen storage disease in adults. Ann Intern Med. 1994 Feb 1;120(3):218-26. doi: 10.7326/0003-4819-120-3-199402010-00008. PMID: 8273986.

12: Dohar JE, Bonilla JA. Processing of adenoid and tonsil specimens in children: a national survey of standard practices and a five-year review of the experience at the Children's Hospital of Pittsburgh. Otolaryngol Head Neck Surg. 1996 Jul;115(1):94-7. doi: 10.1016/S0194-5998(96)70143-2. PMID: 8758637.

13: Kroos MA, Waitfield AE, Joosse M, Winchester B, Reuser AJ, MacDermot KD. A novel acid alpha-glucosidase mutation identified in a Pakistani family with glycogen storage disease type II. J Inherit Metab Dis. 1997 Aug;20(4):556-8. doi: 10.1023/a:1005394706622. PMID: 9266392.

14: Phoenix J, Hopkins P, Bartram C, Beynon RJ, Quinlivan RC, Edwards RH. Effect of vitamin B6 supplementation in McArdle's disease: a strategic case study. Neuromuscul Disord. 1998 May;8(3-4):210-2. doi: 10.1016/s0960-8966(98)00004-2. PMID: 9631404.

15: Burchell A. Glycogen storage diseases and the liver. Baillieres Clin Gastroenterol. 1998 Jun;12(2):337-54. doi: 10.1016/s0950-3528(98)90138-5. PMID: 9890076.

16: Faivre L, Houssin D, Valayer J, Brouard J, Hadchouel M, Bernard O. Long-term outcome of liver transplantation in patients with glycogen storage disease type Ia. J Inherit Metab Dis. 1999 Aug;22(6):723-32. doi: 10.1023/a:1005544117285. PMID: 10472532.

17: Ewert R, Gulijew A, Wensel R, Dandel M, Hummel M, Vogel M, Meyer R, Hetzer R. Die Glykogenose Typ IV als seltene Ursache einer Kardiomyopathie – Bericht einer erfolgreichen Herztransplantation [Glycogenosis type IV as a seldom cause of cardiomyopathy - report about a successful heart transplantation]. Z Kardiol. 1999 Oct;88(10):850-6. German. doi: 10.1007/s003920050361. PMID: 10552189.

18: Corbeel L, Van Lierde S, Jaeken J. Long-term follow-up of portacaval shunt in glycogen storage disease type 1B. Eur J Pediatr. 2000 Apr;159(4):268-72. doi: 10.1007/s004310050068. PMID: 10789932.

19: Kaplan M. Genetic interactions in the pathogenesis of neonatal hyperbilirubinemia: Gilbert's Syndrome and glucose-6-phosphate dehydrogenase deficiency. J Perinatol. 2001 Dec;21 Suppl 1:S30-4; discussion S35-9. doi: 10.1038/sj.jp.7210630. PMID: 11803413.

20: Tsagaraki DP, Evangeliou AE, Tsilimbaris M, Spilioti MG, Mihailidou EP, Lionis C, Pallikaris I. The significance of opthalmologic evaluation in the early diagnosis of inborn errors of metabolism: the Cretan experience. BMC Ophthalmol. 2002 Apr 11;2:2. doi: 10.1186/1471-2415-2-2. PMID: 1950394; PMCID: PMC107746.

21: Kannourakis G. Glycogen storage disease. Semin Hematol. 2002 Apr;39(2):103-6. doi: 10.1053/shem.2002.31920. PMID: 11957192.

22: Enns GM, Packman W. The adolescent with an inborn error of metabolism: medical issues and transition to adulthood. Adolesc Med. 2002 Jun;13(2):315-29, vii. PMID: 11986039.

23: Moses SW. Historical highlights and unsolved problems in glycogen storage disease type 1. Eur J Pediatr. 2002 Oct;161 Suppl 1:S2-9. doi: 10.1007/s00431-002-0997-6. Epub 2002 Aug 23. PMID: 12373565.

24: Hou JW, Wang TR. A 20-year follow-up of a male patient with type Ia glycogen storage disease. Chang Gung Med J. 2003 Apr;26(4):283-7. PMID: 12846528.

25: Haley SM, Fragala MA, Aseltine R, Ni P, Skrinar AM. Development of a disease-specific disability instrument for Pompe disease. Pediatr Rehabil. 2003 Apr-Jun;6(2):77-84. doi: 10.1080/1363849031000139298. PMID: 14534044.

26: Quinlivan R, Beynon RJ. Pharmacological and nutritional treatment for McArdle's disease (Glycogen Storage Disease type V). Cochrane Database Syst Rev. 2004;(3):CD003458. doi: 10.1002/14651858.CD003458.pub2. Update in: Cochrane Database Syst Rev. 2008;(2):CD003458. PMID: 15266486.

27: Hagemans ML, Janssens AC, Winkel LP, Sieradzan KA, Reuser AJ, Van Doorn PA, Van der Ploeg AT. Late-onset Pompe disease primarily affects quality of life in physical health domains. Neurology. 2004 Nov 9;63(9):1688-92. doi: 10.1212/01.wnl.0000142597.69707.78. PMID: 15534256.

28: Ollivier K, Hogrel JY, Gomez-Merino D, Romero NB, Laforêt P, Eymard B, Portero P. Exercise tolerance and daily life in McArdle's disease. Muscle Nerve. 2005 May;31(5):637-41. doi: 10.1002/mus.20251. PMID: 15614801.

29: Hagemans ML, Winkel LP, Van Doorn PA, Hop WJ, Loonen MC, Reuser AJ, Van der Ploeg AT. Clinical manifestation and natural course of late-onset Pompe's disease in 54 Dutch patients. Brain. 2005 Mar;128(Pt 3):671-7. doi: 10.1093/brain/awh384. Epub 2005 Jan 19. PMID: 15659425.

30: Marsden D. Infantile onset Pompe disease: a report of physician narratives from an epidemiologic study. Genet Med. 2005 Feb;7(2):147-50. doi: 10.1097/01.gim.0000154301.76619.5c. PMID: 15714084.

31: Hagemans ML, Winkel LP, Hop WC, Reuser AJ, Van Doorn PA, Van der Ploeg AT. Disease severity in children and adults with Pompe disease related to age and disease duration. Neurology. 2005 Jun 28;64(12):2139-41. doi: 10.1212/01.WNL.0000165979.46537.56. PMID: 15985590.

32: Winkel LP, Hagemans ML, van Doorn PA, Loonen MC, Hop WJ, Reuser AJ, van der Ploeg AT. The natural course of non-classic Pompe's disease; a review of 225 published cases. J Neurol. 2005 Aug;252(8):875-84. doi: 10.1007/s00415-005-0922-9. PMID: 16133732.

33: Muraca M, Burlina AB. Liver and liver cell transplantation for glycogen storage disease type IA. Acta Gastroenterol Belg. 2005 Oct-Dec;68(4):469-72. PMID: 16433006.

34: Hagemans ML, Hop WJ, Van Doorn PA, Reuser AJ, Van der Ploeg AT. Course of disability and respiratory function in untreated late-onset Pompe disease. Neurology. 2006 Feb 28;66(4):581-3. doi: 10.1212/01.wnl.0000198776.53007.2c. PMID: 16505317.

35: Mundy HR, Williams JE, Cousins AJ, Lee PJ. The effect of L-alanine therapy in a patient with adult onset glycogen storage disease type II. J Inherit Metab Dis. 2006 Feb;29(1):226-9. doi: 10.1007/s10545-006-0238-7. PMID: 16601900.

36: Roe CR, Mochel F. Anaplerotic diet therapy in inherited metabolic disease: therapeutic potential. J Inherit Metab Dis. 2006 Apr-Jun;29(2-3):332-40. doi: 10.1007/s10545-006-0290-3. PMID: 16763896.

37: Rommel O, Kley RA, Dekomien G, Epplen JT, Vorgerd M, Hasenbring M. Muscle pain in myophosphorylase deficiency (McArdle's disease): the role of gender, genotype, and pain-related coping. Pain. 2006 Oct;124(3):295-304. doi: 10.1016/j.pain.2006.04.017. Epub 2006 Jun 21. PMID: 16793208.

38: Perez M, Martin MA, Rubio JC, Maté-Muñoz JL, Gómez-Gallego F, Foster C, Andreu AL, Arenas J, Lucia A, Fleck SJ. Exercise capacity in a 78 year old patient with McArdle's disease: it is never too late to start exercising. Br J Sports Med. 2006 Aug;40(8):725-6; discussion 726. doi: 0.1136/bjsm.2006.026666. PMID: 16864568; PMCID: PMC2579473.

39: Bartoli A, Bossù M, Sfasciotti G, Polimeni A. Glycogen Storage Disease type Ib: a paediatric case report. Eur J Paediatr Dent. 2006 Dec;7(4):192-8. PMID: 17168629.

40: Martin AP, Bartels M, Schreiber S, Buehrdel P, Hauss J, Fangmann J. Successful staged kidney and liver transplantation for glycogen storage disease type Ib: A case report. Transplant Proc. 2006 Dec;38(10):3615-9. doi: 10.1016/j.transproceed.2006.10.160. PMID: 17175348.

41: Wagner KR. Enzyme replacement for infantile Pompe disease: the first step toward a cure. Neurology. 2007 Jan 9;68(2):88-9. doi: 10.1212/01.wnl.0000253226.13795.40. PMID: 17210887.

42: Nogales-Gadea G, Arenas J, Andreu AL. Molecular genetics of McArdle's disease. Curr Neurol Neurosci Rep. 2007 Jan;7(1):84-92. doi: 10.1007/s11910-007-0026-2. PMID: 17217859.

43: Belingheri M, Ghio L, Sala A, Menni F, Trespidi L, Ferraresso M, Berardinelli L, Rossi G, Edefonti A, Parini R. Combined liver-kidney transplantation in glycogen storage disease Ia: a case beyond the guidelines. Liver Transpl. 2007 May;13(5):762-4. doi: 10.1002/lt.21147. PMID: 17457869.

44: Hagemans ML, Laforêt P, Hop WJ, Merkies IS, Van Doorn PA, Reuser AJ, Van der Ploeg AT. Impact of late-onset Pompe disease on participation in daily life activities: evaluation of the Rotterdam Handicap Scale. Neuromuscul Disord. 2007 Jul;17(7):537-43. doi: 10.1016/j.nmd.2007.03.006. Epub 2007 May 1. PMID: 17475490.

45: Lee KW, Lee JH, Shin SW, Kim SJ, Joh JW, Lee DH, Kim JW, Park HY, Lee SY, Lee HH, Park JW, Kim SY, Yoon HH, Jung DH, Choe YH, Lee SK. Hepatocyte transplantation for glycogen storage disease type Ib. Cell Transplant. 2007;16(6):629-37. doi: 10.3727/000000007783465019. PMID: 17912954.

46: Quinlivan RM, Beynon RJ. Pharmacological and nutritional treatment trials in McArdle disease. Acta Myol. 2007 Jul;26(1):58-60. PMID: 17915572; PMCID: PMC2949313.

47: Martinuzzi A, Liava A, Trevisi E, Antoniazzi L, Frare M. Chronic therapy for McArdle disease: the randomized trial with ACE inhibitor. Acta Myol. 2007 Jul;26(1):64-6. PMID: 17915574; PMCID: PMC2949319.

48: Martinuzzi A, Liava A, Trevisi E, Frare M, Tonon C, Malucelli E, Manners D, Kemp GJ, Testa C, Barbiroli B, Lodi R. Randomized, placebo-controlled, double-blind pilot trial of ramipril in McArdle's disease. Muscle Nerve. 2008 Mar;37(3):350-7. doi: 10.1002/mus.20937. PMID: 18098237.

49: Storch E, Keeley M, Merlo L, Jacob M, Correia C, Weinstein D. Psychosocial functioning in youth with glycogen storage disease type I. J Pediatr Psychol. 2008 Aug;33(7):728-38. doi: 10.1093/jpepsy/jsn017. Epub 2008 Feb 23. PMID: 18296725.

50: Parini R. Come cambia il paesaggio per le malattie da accumulo lisosomiale [How does the landscape change in lysosomal storage disease]. Pediatr Med Chir. 2007 Sep-Oct;29(5):275-8. Italian. PMID: 18402399.

51: Quinlivan R, Beynon RJ, Martinuzzi A. Pharmacological and nutritional treatment for McArdle disease (Glycogen Storage Disease type V). Cochrane Database Syst Rev. 2008 Apr 16;(2):CD003458. doi: 10.1002/14651858.CD003458.pub3. Update in: Cochrane Database Syst Rev. 2010;(12):CD003458. PMID: 18425888.

52: Nabatame S, Taniike M, Sakai N, Kato-Nishimura K, Mohri I, Kagitani-Shimono K, Okinaga T, Tachibana N, Ozono K. Sleep disordered breathing in childhood-onset acid maltase deficiency. Brain Dev. 2009 Mar;31(3):234-9. doi: 10.1016/j.braindev.2008.03.007. Epub 2008 May 21. PMID: 18495398.

53: van Capelle CI, Winkel LP, Hagemans ML, Shapira SK, Arts WF, van Doorn PA, Hop WC, Reuser AJ, van der Ploeg AT. Eight years experience with enzyme replacement therapy in two children and one adult with Pompe disease. Neuromuscul Disord. 2008 Jun;18(6):447-52. doi: 0.1016/j.nmd.2008.04.009. Epub 2008 May 27. PMID: 18508267.

54: Katzin LW, Amato AA. Pompe disease: a review of the current diagnosis and treatment recommendations in the era of enzyme replacement therapy. J Clin Neuromuscul Dis. 2008 Jun;9(4):421-31. doi: 10.1097/CND.0b013e318176dbe4. PMID: 18525427.

55: Burusnukul P, de Los Reyes EC, Yinger J, Boué DR. Danon disease: an unusual presentation of autism. Pediatr Neurol. 2008 Jul;39(1):52-4. doi:10.1016/j.pediatrneurol.2008.03.011. PMID: 18555174.

56: Kaihara S, Ushigome H, Sakai K, Yoshizawa A, Nobori S, Suzuki T, Okamoto M, Ochiai T, Yoshimura N. Preemptive living donor liver transplantation in glycogen storage disease Ia: case report. Transplant Proc. 2008 Oct;40(8):2815-7. doi: 10.1016/j.transproceed.2008.07.026. PMID: 18929868.

57: Peota C. Intensive care at home. Minn Med. 2008 Oct;91(10):8-9. PMID: 18991005.

58: Hamdan MA, Almalik MH, Mirghani HM. Early administration of enzyme replacement therapy for Pompe disease: short-term follow-up results. J Inherit Metab Dis. 2008 Dec;31 Suppl 2:S431-6. doi: 10.1007/s10545-008-1000-0. Epub 2008 Dec 12. PMID: 19067231.

59: Pascual SI. Phenotype variations in early onset Pompe disease: diagnosis and treatment results with Myozyme. Adv Exp Med Biol. 2009;652:39-46. doi: 10.1007/978-90-481-2813-6_4. PMID: 20225018.

60: Merk T, Wibmer T, Schumann C, Krüger S. Glycogen storage disease type II (Pompe disease)--influence of enzyme replacement therapy in adults. Eur J Neurol. 2009 Feb;16(2):274-7. doi: 10.1111/j.1468-1331.2008.02377.x. Epub 2008 Dec 9. PMID: 19138339.

61: Buehler B. Treatment of adult storage disease: cost versus benefit. Eur J Neurol. 2009 Feb;16(2):159. doi: 10.1111/j.1468-1331.2008.02374.x. PMID: 19146636.

62: Evans S, Shelton F, Holden C, Daly A, Hopkins V, MacDonald A. Monitoring of home safety issues in children on enteral feeds with inherited metabolic disorders. Arch Dis Child. 2010 Sep;95(9):668-72. doi: 10.1136/adc.2008.148338. Epub 2009 Apr 23. PMID: 19395404.

63: Angelini C, Semplicini C, Tonin P, Filosto M, Pegoraro E, Sorarù G, Fanin M. Progress in Enzyme Replacement Therapy in Glycogen Storage Disease Type II. Ther Adv Neurol Disord. 2009 May;2(3):143-53. doi: 10.1177/1756285609103324. PMID: 21179524; PMCID: PMC3002626.

64: Koeberl DD, Kishnani PS, Bali D, Chen YT. Emerging therapies for glycogen storage disease type I. Trends Endocrinol Metab. 2009 Jul;20(5):252-8. doi: 10.1016/j.tem.2009.02.003. Epub 2009 Jun 21. PMID: 19541498.

65: Strothotte S, Strigl-Pill N, Grunert B, Kornblum C, Eger K, Wessig C, Deschauer M, Breunig F, Glocker FX, Vielhaber S, Brejova A, Hilz M, Reiners K, Müller-Felber W, Mengel E, Spranger M, Schoser B. Enzyme replacement therapy with alglucosidase alfa in 44 patients with late-onset glycogen storage disease type 2: 12-month results of an observational clinical trial. J Neurol. 2010

Jan;257(1):91-7. doi: 10.1007/s00415-009-5275-3. Epub 2009 Aug 1. PMID: 19649685.

66: Illés Z, Várdi Visy K. Pompe-kór. II. rész. A betegség enzimpótló kezelése és terápiás megközelítése [Pompe's disease. Part II. Treatment strategies and enzyme replacement]. Ideggyogy Sz. 2009 Sep 30;62(9-10):299-307. Hungarian. PMID: 19835271.

67: Kasahara M, Horikawa R, Sakamoto S, Shigeta T, Tanaka H, Fukuda A, Abe K, Yoshii K, Naiki Y, Kosaki R, Nakagawa A. Living donor liver transplantation for glycogen storage disease type Ib. Liver Transpl. 2009 Dec;15(12):1867-71. doi: 10.1002/lt.21929. PMID: 19938129.

68: Manger B. Lysosomale Speicherkrankheiten [Lysosomal storage diseases]. Z Rheumatol. 2010 Aug;69(6):527-38. German. doi: 10.1007/s00393-010-0627-z. PMID: 20532791.

69: Bernstein DL, Bialer MG, Mehta L, Desnick RJ. Pompe disease: dramatic improvement in gastrointestinal function following enzyme replacement therapy. A report of three later-onset patients. Mol Genet Metab. 2010 Oct-Nov;101(2-3):130-3. doi: 10.1016/j.ymgme.2010.06.003. Epub 2010 Jun 22. PMID: 20638881.

70: Chakrapani A, Vellodi A, Robinson P, Jones S, Wraith JE. Treatment of infantile Pompe disease with alglucosidase alpha: the UK experience. J Inherit Metab Dis. 2010 Dec;33(6):747-50. doi: 10.1007/s10545-010-9206-3. Epub 2010 Sep 24. PMID: 20865334.

71: Boudjemline AM, Isapof A, Witas JB, Petit FM, Gajdos V, Labrune P. Klüver Bucy syndrome following hypoglycaemic coma in a patient with glycogen storage disease type Ib. J Inherit Metab Dis. 2010 Dec;33 Suppl 3:S477-80. doi:10.1007/s10545-010-9243-y. Epub 2010 Nov 20. PMID: 21103936.

72: Quinlivan R, Martinuzzi A, Schoser B. Pharmacological and nutritional treatment for McArdle disease (Glycogen Storage Disease type V). Cochrane Database Syst Rev. 2010 Dec 8;(12):CD003458. doi:10.1002/14651858.CD003458.pub4. Update in: Cochrane Database Syst Rev.2014;11:CD003458. PMID: 21154353.

73: Mancuso M, Orsucci D, Volterrani D, Siciliano G. Cognitive impairment and McArdle disease: Is there a link? Neuromuscul Disord. 2011 May;21(5):356-8. doi:10.1016/j.nmd.2011.02.013. Epub 2011 Mar 5. PMID: 21382715.

74: Tahir A, Malik FR, Ahmad I, Akhtar P. Aetiological factors of chronic liver disease in children. J Ayub Med Coll Abbottabad. 2011 Apr-Jun;23(2):12-4. PMID:24800332.

75: Vielhaber S, Brejova A, Debska-Vielhaber G, Kaufmann J, Feistner H, Schoenfeld MA, Awiszus F. 24-months results in two adults with Pompe disease on enzyme replacement therapy. Clin Neurol Neurosurg. 2011 Jun;113(5):350-7. doi: 10.1016/j.clineuro.2010.09.016. Epub 2011 Apr 7. PMID: 21477922.

76: Kanters TA, Hagemans ML, van der Beek NA, Rutten FF, van der Ploeg AT, Hakkaart L. Burden of illness of Pompe disease in patients only receiving supportive care. J Inherit Metab Dis. 2011 Oct;34(5):1045-52. doi: 10.1007/s10545-011-9320-x. Epub 2011 Apr 16. PMID: 21499718; PMCID: PMC3173621.

77: Kwon JM, Steiner RD. "I'm fine; I'm just waiting for my disease": the new and growing class of presymptomatic patients. Neurology. 2011 Aug 9;77(6):522-3. doi: 10.1212/WNL.0b013e318228c15f. Epub 2011 Jul 13. PMID: 21753177.

78: Yang CC, Chien YH, Lee NC, Chiang SC, Lin SP, Kuo YT, Chen SS, Jong YJ, Hwu WL. Rapid progressive course of later-onset Pompe disease in Chinese patients. Mol Genet Metab. 2011 Nov;104(3):284-8. doi: 10.1016/j.ymgme.2011.06.010. Epub 2011 Jun 22. PMID: 21757382.

79: van Capelle CI, van der Beek NA, de Vries JM, van Doorn PA, Duivenvoorden HJ, Leshner RT, Hagemans ML, van der Ploeg AT. The quick motor function test: a new tool to rate clinical severity and motor function in Pompe patients. J Inherit Metab Dis. 2012 Mar;35(2):317-23. doi: 10.1007/s10545-011-9388-3. Epub 2011 Sep 13. PMID: 21912959; PMCID: PMC3278629.

80: El-Shabrawi MH, Kamal NM. Medical management of chronic liver diseases in children (part I): focus on curable or potentially curable diseases. Paediatr Drugs. 2011 Dec 1;13(6):357-70. doi: 10.2165/11591610-000000000-00000. PMID:21999649.

81: Spiridigliozzi GA, Heller JH, Case LE, Jones HN, Kishnani PS. Early cognitive development in children with infantile Pompe disease. Mol Genet Metab. 2012 Mar;105(3):428-32. doi: 10.1016/j.ymgme.2011.10.012. Epub 2011 Oct 28. PMID: 22217428.

82: Spiridigliozzi GA, Heller JH, Kishnani PS. Cognitive and adaptive functioning of children with infantile Pompe disease treated with enzyme replacement therapy: long-term follow-up. Am J Med Genet C Semin Med Genet. 2012 Feb 15;160C(1):22-9. doi: 10.1002/ajmg.c.31323. Epub 2012 Jan 17. PMID: 22253038.

83: Regnery C, Kornblum C, Hanisch F, Vielhaber S, Strigl-Pill N, Grunert B, Müller-Felber W, Glocker FX, Spranger M, Deschauer M, Mengel E, Schoser B. 36 months observational clinical study of 38 adult Pompe disease patients under alglucosidase alfa enzyme replacement therapy. J Inherit Metab Dis. 2012 Sep;35(5):837-45. doi: 10.1007/s10545-012-9451-8. Epub 2012 Jan 31. PMID: 22290025.

84: Weinreich SS, Rigter T, van El CG, Dondorp WJ, Kostense PJ, van der Ploeg AT, Reuser AJ, Cornel MC, Hagemans ML. Public support for neonatal screening for Pompe disease, a broad-phenotype condition. Orphanet J Rare Dis. 2012 Mar 14;7:15. doi: 10.1186/1750-1172-7-15. PMID: 22413814; PMCID: PMC3351372.

85: Ebbink BJ, Aarsen FK, van Gelder CM, van den Hout JM, Weisglas-Kuperus N, Jaeken J, Lequin MH, Arts WF, van der Ploeg AT. Cognitive outcome of patients with classic infantile Pompe disease receiving enzyme therapy. Neurology. 2012 May 8;78(19):1512-8. doi: 10.1212/WNL.0b013e3182553c11. Epub 2012 Apr 25. PMID: 22539577.

86: Karaki C, Kasahara M, Sakamoto S, Shigeta T, Uchida H, Kanazawa H, Kakiuchi T, Fukuda A, Nakazawa A, Horikawa R, Suzuki Y. Glycemic management in living donor liver transplantation for patients with glycogen storage disease type 1b. Pediatr Transplant. 2012 Aug;16(5):465-70. doi:10.1111/j.1399-3046.2012.01705.x. Epub 2012 May 11. PMID: 22574785.

87: Patel TT, Banugaria SG, Case LE, Wenninger S, Schoser B, Kishnani PS. The impact of antibodies in late-onset Pompe disease: a case series and literature review. Mol Genet Metab. 2012 Jul;106(3):301-9. doi: 10.1016/j.ymgme.2012.04.027. Epub 2012 May 9. PMID: 22613277.

88: Stéphenne X, Debray FG, Smets F, Jazouli N, Sana G, Tondreau T, Menten R, Goffette P, Boemer F, Schoos R, Gersting SW, Najimi M, Muntau AC, Goyens P, Sokal EM. Hepatocyte transplantation using the domino concept in a child with tetrabiopterin nonresponsive phenylketonuria. Cell Transplant. 2012;21(12):2765-70. doi: 10.3727/096368912X653255. Epub 2012 Aug 10. PMID: 22889463.

89: Favejee MM, Huisstede BM, Bussmann JB, Kruijshaar ME, van der Ploeg AT. Physiotherapy management in late-onset Pompe disease: clinical practice in 88 patients. Mol Genet Metab. 2012 Sep;107(1-2):111-5. doi: 10.1016/j.ymgme.2012.07.014. Epub 2012 Jul 20. PMID: 22901700.

90: Toscano A, Schoser B. Enzyme replacement therapy in late-onset Pompe disease: a systematic literature review. J Neurol. 2013 Apr;260(4):951-9. doi: 10.1007/s00415-012-6636-x. Epub 2012 Aug 28. PMID: 22926164.

91: van der Ploeg AT, Barohn R, Carlson L, Charrow J, Clemens PR, Hopkin RJ, Kishnani PS, Laforêt P, Morgan C, Nations S, Pestronk A, Plotkin H, Rosenbloom BE, Sims KB, Tsao E. Open-label extension study following the Late-Onset Treatment Study (LOTS) of alglucosidase alfa. Mol Genet Metab. 2012 Nov;107(3):456-61. doi: 10.1016/j.ymgme.2012.09.015. Epub 2012 Sep 17. PMID: 23031366.

92: Rigter T, Weinreich SS, van El CG, de Vries JM, van Gelder CM, Güngör D, Reuser AJ, Hagemans ML, Cornel MC, van der Ploeg AT. Severely impaired health status at diagnosis of Pompe disease: a cross-sectional analysis to explore the potential utility of neonatal screening. Mol Genet Metab. 2012 Nov;107(3):448-55. doi: 10.1016/j.ymgme.2012.09.017. Epub 2012 Sep 21. PMID: 23040796.

93: Ribes-Koninckx C, Ibars EP, Calzado Agrasot MÁ, Bonora-Centelles A, Miquel BP, Vila Carbó JJ, Aliaga ED, Pallardó JM, Gómez-Lechón MJ, Castell JV. Clinical outcome of hepatocyte transplantation in four pediatric patients with inherited metabolic diseases. Cell Transplant. 2012;21(10):2267-82. doi: 10.3727/096368912X637505. PMID: 23231960.

94: Castro-Jaramillo HE. The cost-effectiveness of enzyme replacement therapy (ERT) for the infantile form of Pompe disease: comparing a high-income country's approach (England) to that of a middle-income one (Colombia). Rev Salud Publica (Bogota). 2012 Jan-Feb;14(1):143-55. doi: 10.1590/s0124-00642012000100012. PMID: 23250322.

95: van der Beek NA, Hagemans ML, van der Ploeg AT, van Doorn PA, Merkies IS. The Rasch-built Pompe-specific activity (R-PAct) scale. Neuromuscul Disord. 2013 Mar;23(3):256-64. doi: 10.1016/j.nmd.2012.10.024. Epub 2012 Dec 28. PMID: 23273871.

96: Kido J, Nakamura K, Matsumoto S, Mitsubuchi H, Ohura T, Shigematsu Y, Yorifuji T, Kasahara M, Horikawa R, Endo F. Current status of hepatic glycogen storage disease in Japan: clinical manifestations, treatments and long-term outcomes. J Hum Genet. 2013 May;58(5):285-92. doi: 10.1038/jhg.2013.17. Epub 2013 Mar 14. PMID: 23486339.

97: Spiridigliozzi GA, Heller JH, Kishnani PS, Van der Ploeg AT, Ebbink BJ, Aarsen FK, van Gelder CM, Van den Hout JM. Cognitive outcome of patients with classic infantile Pompe disease receiving enzyme therapy. Neurology. 2013 Mar 19;80(12):1173. doi: 10.1212/WNL.0b013e31828b8af0. PMID: 23509050.

98: Tsilianidis LA, Fiske LM, Siegel S, Lumpkin C, Hoyt K, Wasserstein M, Weinstein DA. Aggressive therapy improves cirrhosis in glycogen storage disease type IX. Mol Genet Metab. 2013 Jun;109(2):179-82. doi: 10.1016/j.ymgme.2013.03.009. Epub 2013 Mar 21. PMID: 23578772; PMCID: PMC3672367.

99: Ambrosino N, Confalonieri M, Crescimanno G, Vianello A, Vitacca M. The role of respiratory management of Pompe disease. Respir Med. 2013 Aug;107(8):1124-32. doi: 10.1016/j.rmed.2013.03.004. Epub 2013 Apr 12. PMID: 23587901.

100: Borroni B, Cotelli MS, Premi E, Gazzina S, Cosseddu M, Formenti A, Gasparotti R, Filosto M, Padovani A. The brain in late-onset glycogenosis II: a structural and functional MRI study. J Inherit Metab Dis. 2013 Nov;36(6):989-95. doi: 10.1007/s10545-013-9601-7. Epub 2013 Apr 23. PMID: 23609349.

101: Liu AP, Tang WF, Lau ET, Chan KY, Kan AS, Wong KY, Tso WW, Jalal K, Lee SL, Chau CS, Chung BH. Expanded Prader-Willi syndrome due to chromosome 15q11.2-14 deletion: report and a review of literature. Am J Med Genet A. 2013 Jun;161A(6):1309-18. doi: 10.1002/ajmg.a.35909. Epub 2013 Apr 30. PMID: 23633107.

102: Vissing J, Lukacs Z, Straub V. Diagnosis of Pompe disease: muscle biopsy vs blood-based assays. JAMA Neurol. 2013 Jul;70(7):923-7. doi:10.1001/2013.jamaneurol.486. PMID: 23649721.

103: Banka S, Newman WG. A clinical and molecular review of ubiquitous glucose-6-phosphatase deficiency caused by G6PC3 mutations. Orphanet J Rare Dis. 2013 Jun 13;8:84. doi: 10.1186/1750-1172-8-84. PMID: 23758768; PMCID: PMC3718741.

104: Güngör D, Schober AK, Kruijshaar ME, Plug I, Karabul N, Deschauer M, van Doorn PA, van der Ploeg AT, Schoser B, Hanisch F. Pain in adult patients with Pompe disease: a cross-sectional survey. Mol Genet Metab. 2013 Aug;109(4):371-6. doi: 10.1016/j.ymgme.2013.05.021. Epub 2013 Jun 7. PMID: 23849261.

105: Gargiulo M, Herson A, Michon CC, Hogrel JY, Doppler V, Laloui K, Herson S, Payan C, Eymard B, Laforêt P. Attitudes and expectations of patients with neuromuscular diseases about their participation in a clinical trial. Rev Neurol (Paris). 2013 Aug-Sep;169(8-9):670-6. doi: 10.1016/j.neurol.2013.04.005. Epub 2013 Aug 22. PMID: 23972818.

106: Kanters TA, van der Ploeg AT, Brouwer WB, Hakkaart L. The impact of informal care for patients with Pompe disease: an application of the CarerQol instrument. Mol Genet Metab. 2013 Nov;110(3):281-6. doi: 10.1016/j.ymgme.2013.07.020. Epub 2013 Aug 8. PMID: 23973269.

107: Lachmann R, Schoser B. The clinical relevance of outcomes used in late-onset Pompe disease: can we do better? Orphanet J Rare Dis. 2013 Oct 12;8:160. doi: 10.1186/1750-1172-8-160. PMID: 24119230; PMCID: PMC4015278.

108: Maya Aparicio AC, Bernal Bellido C, Tinoco González J, Garcia Ruíz S, Aguilar Romero L, Marín Gómez LM, Suárez Artacho G, Alamo Martínez JM, Serrano Díez-Canedo J, Padillo Ruíz FJ, Gomez Bravo MA. Fifteen years of follow-up of a liver transplant recipient with glycogen storage disease type Ia (Von Gierke disease). Transplant Proc. 2013;45(10):3668-9. doi: 10.1016/j.transproceed.2013.10.033. PMID: 24314991.

109: Sechi A, Deroma L, Paci S, Lapolla A, Carubbi F, Burlina A, Rigoldi M, Di Rocco M. Quality of life in adult patients with glycogen storage disease type I: results of a multicenter italian study. JIMD Rep. 2014;14:47-53. doi: 10.1007/8904_2013_283. Epub 2013 Dec 21. PMID: 24363035; PMCID: PMC4213326.

110: Oguz MM, Aykan E, Yilmaz G, Aytekin C, Karaer K, Açoğlu EA. Glycogen storage disease type 1b: an early onset severe phenotype associated with a novel mutation (IVS4) in the glucose 6-phosphate translocase (SLC37A4) gene in a Turkish patient. Genet Couns. 2014;25(4):389-94. PMID: 25804016.

111: Karabul N, Berndt J, Kornblum C, Kley RA, Wenninger S, Tiling N, Mengel E, Plöckinger U, Vorgerd M, Deschauer M, Schoser B, Hanisch F. Pregnancy and delivery in women with Pompe disease. Mol Genet Metab. 2014 Jun;112(2):148-53. doi: 10.1016/j.ymgme.2014.03.010. Epub 2014 Mar 30. PMID: 24726296.

112: Buckley JP, Quinlivan RM, Sim J, Eston RG, Short DS. Heart rate and perceived muscle pain responses to a functional walking test in McArdle disease. J Sports Sci. 2014;32(16):1561-9. doi: 10.1080/02640414.2014.906045. Epub 2014 Apr 14. PMID: 24731154.

113: Kanters TA, Hoogenboom-Plug I, Rutten-Van Mölken MP, Redekop WK, van der Ploeg AT, Hakkaart L. Cost-effectiveness of enzyme replacement therapy with alglucosidase alfa in classic-infantile patients with Pompe disease. Orphanet J Rare Dis. 2014 May 16;9:75. doi: 10.1186/1750-1172-9-75. PMID: 24884717; PMCID: PMC4038090.

114: Guo CB, Pu CL, Li YC, Zhang MM, Deng Y, Yan LN, Kang Q, Jin XQ. Thirty-five consecutive pediatric living donor liver transplantation: experiences and lessons learned from a single center. Hepatogastroenterology. 2014 Mar-Apr;61(130):391-7. PMID: 24901148.

115: Munguía-Izquierdo D, Santalla A, Lucia A. Cardiorespiratory fitness, physical activity, and quality of life in patients with McArdle disease. Med Sci Sports Exerc. 2015 Apr;47(4):799-808. doi: 10.1249/MSS.0000000000000458. PMID: 25058326.

116: van der Meijden JC, Güngör D, Kruijshaar ME, Muir AD, Broekgaarden HA, van der Ploeg AT. Ten years of the international Pompe survey: patient reported outcomes as a reliable tool for studying treated and untreated children and adults with non-classic Pompe disease. J Inherit Metab Dis. 2015 May;38(3):495-503. doi: 10.1007/s10545-014-9751-2. Epub 2014 Aug 12. PMID: 25112389.

117: van El CG, Rigter T, Reuser AJ, van der Ploeg AT, Weinreich SS, Cornel MC. Newborn screening for pompe disease? a qualitative study exploring professional views. BMC Pediatr. 2014 Aug 14;14:203. doi: 10.1186/1471-2431-14-203. PMID:25124044; PMCID: PMC4139142.

118: Brito-Avô L, Alves JD, Costa JM, Valverde A, Santos L, Araújo F, Aguiar P, Marinho A, Oliveira A, Gomes D. Recomendações para o diagnóstico da forma tardia da doença de Pompe [Diagnosis recommendations for late-onset Pompe disease]. Acta Med Port. 2014 Jul-Aug;27(4):525-9. Portuguese. Epub 2014 Aug 29. PMID: 25203965.

119: Brady S, Godfrey R, Scalco RS, Quinlivan RM. Emotionally-intense situations can result in rhabdomyolysis in McArdle disease. BMJ Case Rep. 2014 Oct 7;2014:bcr2013203272. doi: 10.1136/bcr-2013-203272. PMID: 25293680; PMCID: PMC4187416.

120: Preisler N, Haller RG, Vissing J. Exercise in muscle glycogen storage diseases. J Inherit Metab Dis. 2015 May;38(3):551-63. doi: 10.1007/s10545-014-9771-y. Epub 2014 Oct 18. PMID: 25326273.

121: Kanters TA, Redekop WK, Kruijshaar ME, van der Ploeg AT, Rutten-van Mölken MP, Hakkaart L. Comparison of EQ-5D and SF-6D utilities in Pompe disease. Qual Life Res. 2015 Apr;24(4):837-44. doi: 10.1007/s11136-014-0833-2. Epub 2014 Oct 24. PMID: 25342117.

122: Xiao H, Bian J, Zhang L, Wang Z, Ding A. Gastric cancer following a liver transplantation for glycogen storage disease type Ia (von Gierke disease): A case report. Oncol Lett. 2014 Dec;8(6):2803-2805. doi: 10.3892/ol.2014.2599. Epub 2014 Oct 9. PMID: 25364469; PMCID: PMC4214470.

123: Boentert M, Karabul N, Wenninger S, Stubbe-Dräger B, Mengel E, Schoser B, Young P. Sleep-related symptoms and sleep-disordered breathing in adult Pompe disease. Eur J Neurol. 2015 Feb;22(2):369-76, e27. doi: 10.1111/ene.12582. Epub 2014 Nov 4. PMID: 25367349.

124: Michon CC, Gargiulo M, Hahn-Barma V, Petit F, Nadaj-Pakleza A, Herson A, Eymard B, Labrune P, Laforet P. Cognitive profile of patients with glycogen storage disease type III: a clinical description of seven cases. J Inherit Metab Dis. 2015 May;38(3):573-80. doi: 10.1007/s10545-014-9789-1. Epub 2014 Nov 12. PMID: 25388549.

125: Quinlivan R, Martinuzzi A, Schoser B. Pharmacological and nutritional treatment for McArdle disease (Glycogen Storage Disease type V). Cochrane Database Syst Rev. 2014 Nov 12;2014(11):CD003458. doi: 10.1002/14651858.CD003458.pub5. PMID: 25391139; PMCID: PMC7173724.

126: Tanidir C, Tanidir IC, Tuzcu V. Treatment of depression in an adolescent with cardiomyopathy and arrhythmia. Cardiol Young. 2015 Oct;25(7):1418-20. doi: 10.1017/S1047951114002303. Epub 2014 Nov 17. PMID: 25400066.

127: Favejee MM, van den Berg LE, Kruijshaar ME, Wens SC, Praet SF, Pim Pijnappel WW, van Doorn PA, Bussmann JB, van der Ploeg AT. Exercise training in adults with Pompe disease: the effects on pain, fatigue, and functioning. Arch Phys Med Rehabil. 2015 May;96(5):817-22. doi: 10.1016/j.apmr.2014.11.020. Epub2014 Dec 11. PMID: 25499687.

128: Huber CA, Wunderlich G, Brunn A, Blau T, Fink GR, Lehmann HC. 25-jähriger Patient mit pektanginösen Beschwerden während des muslimischen Fastenmonats [25-year old patient with angina pectoris during religious fasting]. Dtsch Med Wochenschr. 2015 Feb;140(3):202-5. German. doi: 10.1055/s-0041-100076. Epub 2015 Feb 6. PMID: 25658411.

129: Flanagan TB, Sutton JA, Brown LM, Weinstein DA, Merlo LJ. Disordered Eating and Body Esteem Among Individuals with Glycogen Storage Disease. JIMD Rep. 2015;19:23-9. doi: 10.1007/8904_2014_359. Epub 2015 Feb 10. PMID: 25665833; PMCID: PMC4501237.

130: Fiorentino G, Annunziata A, Politano L. Sleep breathing disorders and nocturnal respiratory pattern in patients with glycogenosis type II. Acta Myol. 2014 Oct;33(2):100-3. PMID: 25709380; PMCID: PMC4299164.

131: Hobson-Webb LD, Austin SL, Jain S, Case LE, Greene K, Kishnani PS. Small-fiber neuropathy in pompe disease: first reported cases and prospective screening of a clinic cohort. Am J Case Rep. 2015 Apr 3;16:196-201. doi: 10.12659/AJCR.893309. PMID: 25835646; PMCID: PMC4387956.

132: Burda P, Hochuli M. Hepatic glycogen storage disorders: what have we learned in recent years? Curr Opin Clin Nutr Metab Care. 2015 Jul;18(4):415-21. doi: 10.1097/MCO.0000000000000181. PMID: 26001652.

133: Ross KM, Brown LM, Corrado MM, Chengsupanimit T, Curry LM, Ferrecchia IA, Porras LY, Mathew JT, Weinstein DA. Safety and Efficacy of Chronic Extended Release Cornstarch Therapy for Glycogen Storage Disease Type I. JIMD Rep. 2016;26:85-90. doi: 10.1007/8904_2015_488. Epub 2015 Aug 25. PMID: 26303612; PMCID: PMC4864714.

134: Mattosova S, Hlavata A, Spalek P, Kotysova L, Macekova D, Chandoga J. Late onset form of Pompe disease. Bratisl Lek Listy. 2015;116(8):502-5. doi: 10.4149/bll_2015_097. PMID: 26350092.

135: Kanters TA, Redekop WK, Rutten-Van Mölken MP, Kruijshaar ME, Güngör D, van der Ploeg AT, Hakkaart L. A conceptual disease model for adult Pompe disease. Orphanet J Rare Dis. 2015 Sep 15;10:112. doi: 10.1186/s13023-015-0334-6. PMID: 26374742; PMCID: PMC4570629.

136: MENA Pompe Working Group, Al Jasmi F, Al Jumah M, Alqarni F, Al-Sanna'a N, Al-Sharif F, Bohlega S, Cupler EJ, Fathalla W, Hamdan MA, Makhseed N, Nafissi S, Nilipour Y, Selim L, Shembesh N, Sunbul R, Tonekaboni SH. Diagnosis and treatment of late-onset Pompe disease in the Middle East and North Africa region: consensus recommendations from an expert group. BMC Neurol. 2015 Oct 15;15:205. doi: 10.1186/s12883-015-0412-3. PMID: 26471939; PMCID: PMC4608291.

137: Güngör D, Kruijshaar ME, Plug I, Rizopoulos D, Kanters TA, Wens SC, Reuser AJ, van Doorn PA, van der Ploeg AT. Quality of life and participation in daily life of adults with Pompe disease receiving enzyme replacement therapy: 10 years of international follow-up. J Inherit Metab Dis. 2016 Mar;39(2):253-60. doi: 10.1007/s10545-015-9889-6. Epub 2015 Nov 3. PMID: 26531313; PMCID: PMC4754323.

138: Yang CF, Yang CC, Liao HC, Huang LY, Chiang CC, Ho HC, Lai CJ, Chu TH, Yang TF, Hsu TR, Soong WJ, Niu DM. Very Early Treatment for Infantile-Onset Pompe Disease Contributes to Better Outcomes. J Pediatr. 2016 Feb;169:174-80.e1. doi: 10.1016/j.jpeds.2015.10.078. Epub 2015 Dec 10. PMID: 26685070.

139: Liu Y, Yang Y, Wang B, Wu L, Liang H, Kan Q, Cao Z, Zhao Y, Zhou X. Infantile Pompe disease: A case report and review of the Chinese literature. Exp Ther Med. 2016 Jan;11(1):235-238. doi: 10.3892/etm.2015.2862. Epub 2015 Nov 12. PMID: 26889246; PMCID: PMC4726885.

140: Melis D, Rossi A, Pivonello R, Del Puente A, Pivonello C, Cangemi G, Negri M, Colao A, Andria G, Parenti G. Reduced bone mineral density in glycogen storage disease type III: evidence for a possible connection between metabolic imbalance and bone homeostasis. Bone. 2016 May;86:79-85. doi: 10.1016/j.bone.2016.02.012. Epub 2016 Feb 23. PMID: 26924264.

141: Brown A, Crowe L, Boneh A, Anderson V. Parent Coping and the Behavioural and Social Outcomes of Children Diagnosed with Inherited Metabolic Disorders. JIMD Rep. 2017;31:29-36. doi: 10.1007/8904_2016_544. Epub 2016 Mar 24. PMID: 27008193; PMCID: PMC5388638.

142: Aslan GK, Huseyinsinoglu BE, Oflazer P, Gurses N, Kiyan E. Inspiratory Muscle Training in Late-Onset Pompe Disease: The Effects on Pulmonary Function Tests, Quality of Life, and Sleep Quality. Lung. 2016 Aug;194(4):555-61. doi: 10.1007/s00408-016-9881-4. Epub 2016 Apr 22. PMID: 27106274.

143: Garcia MJ. Constrictive Pericarditis Versus Restrictive Cardiomyopathy? J Am Coll Cardiol. 2016 May 3;67(17):2061-76. doi: 10.1016/j.jacc.2016.01.076. PMID: 27126534.

144: Sugie K, Yoshizawa H, Onoue K, Nakanishi Y, Eura N, Ogawa M, Nakano T, Sakaguchi Y, Hayashi YK, Kishimoto T, Shima M, Saito Y, Nishino I, Ueno S. Early onset of cardiomyopathy and intellectual disability in a girl with Danon disease associated with a de novo novel mutation of the LAMP2 gene. Neuropathology. 2016 Dec;36(6):561-565. doi: 10.1111/neup.12307. Epub 2016 May 5. PMID: 27145725.

145: Kumbar V, Simha J, Gundappa P. Anaesthetic management of a patient with Pompe disease for kyphoscoliosis correction. Indian J Anaesth. 2016 May;60(5):349-51. doi: 10.4103/0019-5049.181597. PMID: 27212723; PMCID: PMC4870949.

146: Boentert M, Dräger B, Glatz C, Young P. Sleep-Disordered Breathing and Effects of Noninvasive Ventilation in Patients with Late-Onset Pompe Disease. J Clin Sleep Med. 2016 Dec 15;12(12):1623-1632. doi: 10.5664/jcsm.6346. PMID: 27568896; PMCID: PMC5155202.

147: Lisi EC, Gillespie S, Laney D, Ali N. Patients' perspectives on newborn screening for later-onset lysosomal storage diseases. Mol Genet Metab. 2016 Sep;119(1-2):109-14. doi: 10.1016/j.ymgme.2016.07.009. Epub 2016 Jul 21. PMID:27591925.

148: Wilson RD, De Bie I, Armour CM, Brown RN, Campagnolo C, Carroll JC, Okun N, Nelson T, Zwingerman R, Audibert F, Brock JA, Brown RN, Campagnolo C, Carroll JC, De Bie I, Johnson JA, Okun N, Pastruck M, Vallée-Pouliot K, Wilson RD, Zwingerman R, Armour C, Chitayat D, De Bie I, Fernandez S, Kim R, Lavoie J, Leonard N, Nelson T, Taylor S, Van Allen M, Van Karnebeek C. Joint SOGC-CCMG Opinion for Reproductive Genetic Carrier Screening: An Update for All Canadian Providers of Maternity and Reproductive Healthcare in the Era of Direct-to-Consumer Testing. J Obstet Gynaecol Can. 2016 Aug;38(8):742-762.e3. doi: 10.1016/j.jogc.2016.06.008. PMID: 27638987.

149: Gupta S. Screening: Baby's first test. Nature. 2016 Sep 22;537(7621):S162-4. doi: 10.1038/537S162a. PMID: 27652786.

150: Lai CJ, Hsu TR, Yang CF, Chen SJ, Chuang YC, Niu DM. Cognitive Development in Infantile-Onset Pompe Disease Under Very Early Enzyme Replacement Therapy. J Child Neurol. 2016 Dec;31(14):1617-1621. doi: 10.1177/0883073816665549. Epub 2016 Sep 21. PMID: 27655474.

151: Chu YP, Sheng B, Lau KK, Chan HF, Kam GY, Lee HH, Mak CM. Clinical manifestation of late onset Pompe disease patients in Hong Kong. Neuromuscul Disord. 2016 Dec;26(12):873-879. doi: 10.1016/j.nmd.2016.09.004. Epub 2016 Sep 13. PMID: 27692865.

152: Figueroa-Bonaparte S, Segovia S, Llauger J, Belmonte I, Pedrosa I, Alejaldre A, Mayos M, Suárez-Cuartín G, Gallardo E, Illa I, Díaz-Manera J; Spanish Pompe Study Group. Muscle MRI Findings in Childhood/Adult Onset Pompe Disease Correlate with Muscle Function. PLoS One. 2016 Oct 6;11(10):e0163493. doi: 10.1371/journal.pone.0163493. PMID: 27711114; PMCID: PMC5053479.

153: Boentert M, Prigent H, Várdi K, Jones HN, Mellies U, Simonds AK, Wenninger S, Barrot Cortés E, Confalonieri M. Practical Recommendations for Diagnosis and Management of Respiratory Muscle Weakness in Late-Onset Pompe Disease. Int J Mol Sci. 2016 Oct 17;17(10):1735. doi: 10.3390/ijms17101735. PMID: 27763517; PMCID: PMC5085764.

154: Blöß S, Klemann C, Rother AK, Mehmecke S, Schumacher U, Mücke U, Mücke M, Stieber C, Klawonn F, Kortum X, Lechner W, Grigull L. Diagnostic needs for rare diseases and shared prediagnostic phenomena: Results of a German-wide expert Delphi survey. PLoS One. 2017 Feb 24;12(2):e0172532. doi: 10.1371/journal.pone.0172532. PMID: 28234950; PMCID: PMC5325301.

155: van der Ploeg AT, Kruijshaar ME, Toscano A, Laforêt P, Angelini C, Lachmann RH, Pascual Pascual SI, Roberts M, Rösler K, Stulnig T, van Doorn PA, Van den Bergh PYK, Vissing J, Schoser B; European Pompe Consortium. European consensus for starting and stopping enzyme replacement therapy in adult patients with Pompe disease: a 10-year experience. Eur J Neurol. 2017 Jun;24(6):768-e31. doi: 10.1111/ene.13285. Epub 2017 May 6. PMID: 28477382.

156: Spiridigliozzi GA, Keeling LA, Stefanescu M, Li C, Austin S, Kishnani PS. Cognitive and academic outcomes in long-term survivors of infantile-onset Pompe disease: A longitudinal follow-up. Mol Genet Metab. 2017 Jun;121(2):127-137. doi: 10.1016/j.ymgme.2017.04.014. Epub 2017 May 1. PMID: 28495044; PMCID: PMC5985833.

157: Yardeni M, Weisman O, Mandel H, Weinberger R, Quarta G, Salazar-Mendiguchía J, Garcia-Pavia P, Lobato-Rodríguez MJ, Simon LF, Dov F, Arad M, Gothelf D. Psychiatric and cognitive characteristics of individuals with Danon disease (LAMP2 gene mutation). Am J Med Genet A. 2017 Sep;173(9):2461-2466. doi: 10.1002/ajmg.a.38320. Epub 2017 Jun 19. PMID: 28627787; PMCID: PMC5561494.

158: Scalco RS, Morrow JM, Booth S, Chatfield S, Godfrey R, Quinlivan R. Misdiagnosis is an important factor for diagnostic delay in McArdle disease. Neuromuscul Disord. 2017 Sep;27(9):852-855. doi: 10.1016/j.nmd.2017.04.013. Epub 2017 May 3. PMID: 28629675.

159: Kumar S, Kumar A. Unusual Presentation of Atypical Infantile Pompe Disease in the Newborn Period with Left Ventricular Hypertrophy. J Clin Diagn Res. 2017 May;11(5):SD01-SD02. doi: 10.7860/JCDR/2017/20756.9849. Epub 2017 May 1. PMID: 28658866; PMCID: PMC5483768.

160: Raza M, Arif F, Giyanwani PR, Azizullah S, Kumari S. Dietary Therapy for Von Gierke's Disease: A Case Report. Cureus. 2017 Aug 8;9(8):e1548. doi: 10.7759/cureus.1548. PMID: 29018645; PMCID: PMC5630462.

161: Wehbe TW, Abi Chahine NH, Annous AA, Ferri MA, Boulous RC, El-Mestrah MF. A case report of congenital glycogen storage liver cirrhosis treated with bone marrow derived stem cells. Stem Cell Investig. 2017 Sep 7;4:73. doi: 10.21037/sci.2017.07.04. PMID: 29057245; PMCID: PMC5638994.

162: Minter Baerg MM, Stoway SD, Hart J, Mott L, Peck DS, Nett SL, Eckerman JS, Lacey JM, Turgeon CT, Gavrilov D, Oglesbee D, Raymond K, Tortorelli S, Matern D, Mørkrid L, Rinaldo P. Precision newborn screening for lysosomal disorders. Genet Med. 2018 Aug;20(8):847-854. doi: 10.1038/gim.2017.194. Epub 2017 Nov 9. PMID: 29120458.

163: Schoser B, Bilder DA, Dimmock D, Gupta D, James ES, Prasad S. The humanistic burden of Pompe disease: are there still unmet needs? A systematic review. BMC Neurol. 2017 Nov 22;17(1):202. doi: 10.1186/s12883-017-0983-2. PMID: 29166883; PMCID: PMC5700516.

164: Rousseau-Nepton I, Huot C, Laforte D, Mok E, Fenyves D, Constantin E, Mitchell J. Sleep and quality of life of patients with glycogen storage disease on standard and modified uncooked cornstarch. Mol Genet Metab. 2018 Mar;123(3):326-330. doi: 10.1016/j.ymgme.2017.09.003. Epub 2017 Sep 11. PMID: 29223626.

165: Kanters TA, van der Ploeg AT, Kruijshaar ME, Rizopoulos D, Redekop WK, Rutten-van Mӧlken MPMH, Hakkaart-van Roijen L. Cost-effectiveness of enzyme replacement therapy with alglucosidase alfa in adult patients with Pompe disease. Orphanet J Rare Dis. 2017 Dec 13;12(1):179. doi: 10.1186/s13023-017-0731-0. PMID: 29237491; PMCID: PMC5729274.

166: Rodríguez-Gómez I, Santalla A, Díez-Bermejo J, Munguía-Izquierdo D, Alegre LM, Nogales-Gadea G, Arenas J, Martín MÁ, Lucía A, Ara I. A New Condition in McArdle Disease: Poor Bone Health-Benefits of an Active Lifestyle. Med Sci Sports Exerc. 2018 Jan;50(1):3-10. doi: 10.1249/MSS.0000000000001414. PMID: 29251685.

167: Desai AK, Walters CK, Cope HL, Kazi ZB, DeArmey SM, Kishnani PS. Enzyme replacement therapy with alglucosidase alfa in Pompe disease: Clinical experience with rate escalation. Mol Genet Metab. 2018 Feb;123(2):92-96. doi:10.1016/j.ymgme.2017.12.435. Epub 2017 Dec 23. PMID: 29289479; PMCID:PMC5808871.

168: Carnero-Gregorio M, Molares-Vila A, Corbalán-Rivas A, Villaverde-Taboada C, Rodríguez-Cerdeira C. Effect of VSL#3 Probiotic in a Patient with Glycogen Storage Disease Type Ia and Irritable Bowel Disease-like Disease. Probiotics Antimicrob Proteins. 2019 Mar;11(1):143-149. doi: 10.1007/s12602-017-9372-9. PMID: 29441457.

169: Ebbink BJ, Poelman E, Aarsen FK, Plug I, Régal L, Muentjes C, van der Beek NAME, Lequin MH, van der Ploeg AT, van den Hout JMP. Classic infantile Pompe patients approaching adulthood: a cohort study on consequences for the brain. Dev Med Child Neurol. 2018 Jun;60(6):579-586. doi: 10.1111/dmcn.13740. Epub 2018 Mar 24. PMID: 29573408.

170: Pruniski B, Lisi E, Ali N. Newborn screening for Pompe disease: impact on families. J Inherit Metab Dis. 2018 Nov;41(6):1189-1203. doi: 10.1007/s10545-018-0159-2. Epub 2018 Mar 28. PMID: 29594646.

171: Ricci G, Baldanzi S, Seidita F, Proietti C, Carlini F, Peviani S, Antonini G, Vianello A, Siciliano G; Italian GSD II group. A mobile app for patients with Pompe disease and its possible clinical applications. Neuromuscul Disord. 2018 Jun;28(6):471-475. doi: 10.1016/j.nmd.2018.03.005. Epub 2018 Mar 12. PMID: 29655528.

172: Dow DE, Seed PC. Clostridium difficile cure with fecal microbiota transplantation in a child with Pompe disease: a case report. J Med Case Rep. 2018 Apr 28;12(1):112. doi: 10.1186/s13256-018-1659-2. PMID: 29703246; PMCID: PMC5924470.

173: Owens P, Wong M, Bhattacharya K, Ellaway C. Infantile-onset Pompe disease: A case series highlighting early clinical features, spectrum of disease severity and treatment response. J Paediatr Child Health. 2018 Nov;54(11):1255-1261. doi: 10.1111/jpc.14070. Epub 2018 Jun 11. PMID: 29889338.

174: Chan EK, Kornberg AJ. Elevated Creatine Kinase in a 6-Year-Old Boy. Semin Pediatr Neurol. 2018 Jul;26:46-49. doi: 10.1016/j.spen.2017.03.003. Epub 2017 Apr 1. PMID: 29961517.

175: Figueroa-Bonaparte S, Llauger J, Segovia S, Belmonte I, Pedrosa I, Montiel E, Montesinos P, Sánchez-González J, Alonso-Jiménez A, Gallardo E, Illa I; Spanish Pompe group, Díaz-Manera J. Quantitative muscle MRI to follow up late onset Pompe patients: a prospective study. Sci Rep. 2018 Jul 18;8(1):10898. doi: 10.1038/s41598-018-29170-7. PMID: 30022036; PMCID: PMC6052002.

176: Steunenberg TAH, Peeks F, Hoogeveen IJ, Mitchell JJ, Mundy H, de Boer F, Lubout CMA, de Souza CF, Weinstein DA, Derks TGJ. Safety issues associated with dietary management in patients with hepatic glycogen storage disease. Mol Genet Metab. 2018 Sep;125(1-2):79-85. doi: 10.1016/j.ymgme.2018.07.004. Epub 2018 Jul 18. PMID: 30037503.

177: Kouwenberg CV, Voermans NC, Quinlivan R, van den Engel-Hoek L. Mastication and Oral Motor Function in McArdle Disease: Patient Reported Complaints. J Neuromuscul Dis. 2018;5(3):353-357. doi: 10.3233/JND-180320. PMID: 30103350.

178: Michelson J. My Diagnostic Odyssey-A Call to Expand Access to Genomic Testing for the Next Generation. Hastings Cent Rep. 2018 Jul;48 Suppl 2:S32-S34. doi: 10.1002/hast.882. PMID: 30133734.

179: Scheidegger O, Leupold D, Sauter R, Findling O, Rösler KM, Hundsberger T. 36-Months follow-up assessment after cessation and resuming of enzyme replacement therapy in late onset Pompe disease: data from the Swiss Pompe Registry. J Neurol. 2018 Dec;265(12):2783-2788. doi: 10.1007/s00415-018-9065-7. Epub 2018 Sep 19. PMID: 30232608.

180: Martinez CC, Tonon T, Nalin T, Refosco LF, de Souza CFM, Schwartz IVD. Feeding Difficulties and Orofacial Myofunctional Disorder in Patients with Hepatic Glycogen Storage Diseases. JIMD Rep. 2019;45:21-27. doi: 10.1007/8904_2018_131. Epub 2018 Sep 22. PMID: 30242630; PMCID: PMC6336547.

181: Musumeci O, Marino S, Granata F, Morabito R, Bonanno L, Brizzi T, Lo Buono V, Corallo F, Longo M, Toscano A. Central nervous system involvement in late-onset Pompe disease: clues from neuroimaging and neuropsychological analysis. Eur J Neurol. 2019 Mar;26(3):442-e35. doi: 10.1111/ene.13835. Epub 2018 Nov 15. PMID: 30312517.

182: Sugie K, Komaki H, Eura N, Shiota T, Onoue K, Tsukaguchi H, Minami N, Ogawa M, Kiriyama T, Kataoka H, Saito Y, Nonaka I, Nishino I. A Nationwide Survey on Danon Disease in Japan. Int J Mol Sci. 2018 Nov 8;19(11):3507. doi: 10.3390/ijms19113507. PMID: 30413001; PMCID: PMC6274850.

183: Wenninger S, Greckl E, Babačić H, Stahl K, Schoser B. Safety and efficacy of short- and long-term inspiratory muscle training in late-onset Pompe disease (LOPD): a pilot study. J Neurol. 2019 Jan;266(1):133-147. doi: 10.1007/s00415-018-9112-4. Epub 2018 Nov 14. PMID: 30430231.

184: Kaiser N, Gautschi M, Bosanska L, Meienberg F, Baumgartner MR, Spinas GA, Hochuli M. Glycemic control and complications in glycogen storage disease type I: Results from the Swiss registry. Mol Genet Metab. 2019 Apr;126(4):355-361. doi: 10.1016/j.ymgme.2019.02.008. Epub 2019 Feb 28. PMID: 30846352.

185: Iolascon G, Vitacca M, Carraro E, Chisari C, Fiore P, Messina S, Mongini TEG, Sansone VA, Toscano A, Siciliano G. The role of rehabilitation in the management of late-onset Pompe disease: a narrative review of the level of evidence. Acta Myol. 2018 Dec 1;37(4):241-251. PMID: 30944902; PMCID: PMC6416696.

186: Berger KI, Kanters S, Jansen JP, Stewart A, Sparks S, Haack KA, Bolzani A, Siliman G, Hamed A. Forced vital capacity and cross-domain late-onset Pompe disease outcomes: an individual patient-level data meta-analysis. J Neurol. 2019 Sep;266(9):2312-2321. doi: 10.1007/s00415-019-09401-1. Epub 2019 Jun 11. PMID: 31187190; PMCID: PMC6687674.

187: Dunne TF, Geberhiwot T, Jones R. Acute psychosis in glycogen storage disease: a rare but severe complication. BMJ Case Rep. 2019 Jul 4;12(7):e222307. doi: 10.1136/bcr-2017-222307. PMID: 31272990; PMCID: PMC6613960.

188: Jones HN, Kuchibhatla M, Crisp KD, Hobson Webb LD, Case L, Batten MT, Marcus JA, Kravitz RM, Kishnani PS. Respiratory muscle training (RMT) in late-onset Pompe disease (LOPD): A protocol for a sham-controlled clinical trial. Mol Genet Metab. 2019 Aug;127(4):346-354. doi: 10.1016/j.ymgme.2019.05.001. Epub 2019 May 8. PMID: 31303277; PMCID: PMC6717661.

189: Francini-Pesenti F, Tresso S, Vitturi N. Modified Atkins ketogenic diet improves heart and skeletal muscle function in glycogen storage disease type III. Acta Myol. 2019 Mar 1;38(1):17-20. PMID: 31309177; PMCID: PMC6598403.

190: Bay LB, Denzler I, Durand C, Eiroa H, Frabasil J, Fainboim A, Maxit C, Schenone A, Spécola N. Infantile-onset Pompe disease: Diagnosis and management. Arch Argent Pediatr. 2019 Aug 1;117(4):271-278. English, Spanish. doi: 10.5546/aap.2019.eng.271. PMID: 31339275.

191: Grassi B, Porcelli S, Marzorati M. Translational Medicine: Exercise Physiology Applied to Metabolic Myopathies. Med Sci Sports Exerc. 2019 Nov;51(11):2183-2192. doi: 10.1249/MSS.0000000000002056. PMID: 31634290.

192: Artime E, Shui I, Mendez I, Tcherny-Lessenot S, D'Arbigny P, Alfaro N, Qizilbash N. Pre/post effectiveness evaluation of updated additional risk minimisation measures for an orphan disease: Myozyme (alglucosidase alfa) Safety Information Packet. Pharmacoepidemiol Drug Saf. 2020 Jan;29(1):103-110. doi: 10.1002/pds.4905. Epub 2019 Oct 31. PMID: 31667955.

193: Iolascon G, Vitacca M, Carraro E, Chisari C, Fiore P, Messina S, Mongini T, Moretti A, Sansone VA, Toscano A, Siciliano G; AIM (Italian Association of Myology), AIPO (Italian Association of Hospital Pulmonologists), SIRN (Italian Society of Neurorehabilitation), and SIMFER (Italian Society of Physical

Medicine and Rehabilitation). Adapted physical activity and therapeutic exercise in late-onset Pompe disease (LOPD): a two-step rehabilitative approach. Neurol Sci. 2020 Apr;41(4):859-868. doi: 10.1007/s10072-019-04178-7. Epub 2019 Dec 7. PMID: 31811531.

194: Wicker C, Roda C, Perry A, Arnoux JB, Brassier A, Castelle M, Servais A, Donadieu J, Bouchereau J, Pigneur B, Labrune P, Ruemmele FM, de Lonlay P. Infectious and digestive complications in glycogen storage disease type Ib: Study of a French cohort. Mol Genet Metab Rep. 2020 Apr 9;23:100581. doi: 10.1016/j.ymgmr.2020.100581. PMID: 32300528; PMCID: PMC7152669.

195: Kuchenbecker KS, Kirschner-Hermanns R, Kornblum C, Jaekel A, Anding R, Kohler A. Urodynamic and clinical studies in patients with late-onset Pompe disease and lower urinary tract symptoms. Neurourol Urodyn. 2020 Jun;39(5):1437-1446. doi: 10.1002/nau.24369. Epub 2020 Apr 28. PMID: 32343026.

196: Korlimarla A, Spiridigliozzi GA, Crisp K, Herbert M, Chen S, Malinzak M, Stefanescu M, Austin SL, Cope H, Zimmerman K, Jones H, Provenzale JM, Kishnani PS. Novel approaches to quantify CNS involvement in children with Pompe disease. Neurology. 2020 Aug 11;95(6):e718-e732. doi: 10.1212/WNL.0000000000009979. Epub 2020 Jun 9. PMID: 32518148; PMCID: PMC7455359.

197: Rodríguez-Gómez I, Santalla A, Diez-Bermejo J, Munguía-Izquierdo D, Alegre LM, Nogales-Gadea G, Arenas J, Martín MA, Lucia A, Ara I. Sex Differences and the Influence of an Active Lifestyle on Adiposity in Patients with McArdle Disease. Int J Environ Res Public Health. 2020 Jun 17;17(12):4334. doi: 10.3390/ijerph17124334. PMID: 32560448; PMCID: PMC7344565.

198: Marusic T, Zerjav Tansek M, Sirca Campa A, Mezek A, Berden P, Battelino T, Groselj U. Normalization of obstructive cardiomyopathy and improvement of hepatopathy on ketogenic diet in patient with glycogen storage disease (GSD) type IIIa. Mol Genet Metab Rep. 2020 Jul 16;24:100628. doi: 10.1016/j.ymgmr.2020.100628. PMID: 32714838; PMCID: PMC7371897.

199: Scalco RS, Stemmerik M, Løkken N, Vissing CR, Madsen KL, Michalak Z, Pattni J, Godfrey R, Samandouras G, Bassett P, Holton JL, Krag T, Haller RG, Sewry C, Wigley R, Vissing J, Quinlivan R. Results of an open label feasibility study of sodium valproate in people with McArdle disease. Neuromuscul Disord. 2020 Sep;30(9):734-741. doi: 10.1016/j.nmd.2020.04.009. Epub 2020 May 16. PMID: 32811700.

200: Goker-Alpan O, Kasturi VG, Sohi MK, Limgala RP, Austin SL, Jennelle T, Banikazemi M, Kishnani PS. Pregnancy Outcomes in Late Onset Pompe Disease. Life (Basel). 2020 Sep 11;10(9):194. doi: 10.3390/life10090194. PMID: 32932790; PMCID: PMC7556025.

201: Strokova TV, Bagaeva ME, Zubovich AI, Pavlovskaya EV, Taran NN, Tin IF, Matinyan IA, Dremucheva TA, Kutyreva EA, Vasil'eva EA. [Nutrition and orphan diseases]. Vopr Pitan. 2020;89(4):193-202. Russian. doi: 10.24411/0042-8833-2020-10053. Epub 2020 Jul 29. PMID: 32986332.

202: Pinós T, Andreu AL, Bruno C, Hadjigeorgiou GM, Haller RG, Laforêt P, Lucía A, Martín MA, Martinuzzi A, Navarro C, Oflazer P, Pouget J, Quinlivan R, Sacconi S, Scalco RS, Toscano A, Vissing J, Vorgerd M, Wakelin A, Martí R; EUROMAC Consortium. Creation and implementation of a European registry for patients with McArdle disease and other muscle glycogenoses (EUROMAC registry). Orphanet J Rare Dis. 2020 Oct 15;15(1):187. doi: 10.1186/s13023-020-01455-z. PMID: 33054807; PMCID: PMC7558742.

203: Sawada T, Kido J, Nakamura K. Newborn Screening for Pompe Disease. Int J Neonatal Screen. 2020 Apr 5;6(2):31. doi: 10.3390/ijns6020031. PMID: 33073027; PMCID: PMC7423004.

204: Shah NM, Sharma L, Ganeshamoorthy S, Kaltsakas G. Respiratory failure and sleep-disordered breathing in late-onset Pompe disease: a narrative review. J Thorac Dis. 2020 Oct;12(Suppl 2):S235-S247. doi: 10.21037/jtd-cus-2020-007. PMID: 33214927; PMCID: PMC7642632.

205: Gao Q, Wang S, Ren J, Wen X. Measuring parent proxy-reported quality of life of 11 rare diseases in children in Zhejiang, China. Health Qual Life Outcomes. 2020 Nov 23;18(1):372. doi: 10.1186/s12955-020-01572-0. PMID: 33225969; PMCID: PMC7682005.

206: Galeotti A, De Rosa S, Uomo R, Dionisi-Vici C, Deodato F, Taurisano R, Olivieri G, Festa P. Orofacial features and pediatric dentistry in the long-term management of Infantile Pompe Disease children. Orphanet J Rare Dis. 2020 Nov 23;15(1):329. doi: 10.1186/s13023-020-01615-1. PMID: 33228748; PMCID: PMC7685588.

207: Mengel E, Gaedeke J, Gothe H, Krupka S, Lachmann A, Reinke J, Ohlmeier C. The patient journey of patients with Fabry disease, Gaucher disease and Mucopolysaccharidosis type II: A German-wide telephone survey. PLoS One. 2020 Dec 31;15(12):e0244279. doi: 10.1371/journal.pone.0244279. PMID: 33382737; PMCID: PMC7775043.

208: Della Pepa G, Vetrani C, Lupoli R, Massimino E, Lembo E, Riccardi G, Capaldo B. Uncooked cornstarch for the prevention of hypoglycemic events. Crit Rev Food Sci Nutr. 2021 Jan 18:1-14. doi: 10.1080/10408398.2020.1864617. Epub ahead of print. PMID: 33455416.

209: Jorge NB, Tommaso AMA, Hessel G. ANTHROPOMETRIC AND DIETARY ASSESSMENT OF PATIENTS WITH GLYCOGENOSIS TYPE I. Rev Paul Pediatr. 2021 Feb 5;39:e2020046. doi: 10.1590/1984-0462/2021/39/2020046. PMID: 33566881; PMCID: PMC7875543.

210: Liu M, Sun LY. Liver Transplantation for Glycogen Storage Disease Type IV. Front Pediatr. 2021 Feb 19;9:633822. doi: 10.3389/fped.2021.633822. PMID: 33681109; PMCID: PMC7933444.
